# Development and validation of a digital biomarker predicting acute kidney injury following cardiac surgery on an hourly basis

**DOI:** 10.1016/j.xjon.2023.09.023

**Published:** 2023-09-23

**Authors:** Linda Lapp, Marc Roper, Kimberley Kavanagh, Stefan Schraag

**Affiliations:** a Department of Computer and Information Sciences, Faculty of Science, University of Strathclyde, Glasgow, Scotland; b Department of Mathematics and Statistics, Faculty of Science, University of Strathclyde, Glasgow, Scotland; c Department of Anaesthesia and Perioperative Medicine, Golden Jubilee National Hospital, Clydebank, United Kingdom

**Keywords:** cardiac surgery, acute kidney injury, intensive care unit, prediction model, biomarkers, risk prediction

## Abstract

**Objectives:**

To develop and validate a digital biomarker for predicting the onset of acute kidney injury (AKI) on an hourly basis up to 24 hours in advance in the intensive care unit after cardiac surgery.

**Methods:**

The study analyzed data from 6056 adult patients undergoing coronary artery bypass graft and/or valve surgery between April 1, 2012, and December 31, 2018 (development phase, training, and testing) and 3572 patients between January 1, 2019, and June 30, 2022 (validation phase). The study used 2 dynamic predictive modeling approaches, namely logistic regression and bootstrap aggregated regression trees machine (BARTm), to predict AKI. The mean area under the receiver operating characteristic curve (AUC), sensitivity, specificity, and positive and negative predictive values across all lead times before the occurrence of AKI were reported. The clinical practicality was assessed using calibration.

**Results:**

Of all included patients, 8.45% and 16.66% had AKI in the development and validation phases, respectively. When applied to testing data, AKI was predicted with the mean AUC of 0.850 and 0.802 by BARTm and logistic regression, respectively. When applied to validation data, BARTm and LR resulted in a mean AUC of 0.844 and 0.786, respectively.

**Conclusions:**

This study demonstrated the successful prediction of AKI on an hourly basis up to 24 hours in advance. The digital biomarkers developed and validated in this study have the potential to assist clinicians in optimizing treatment and implementing preventive strategies for patients at risk of developing AKI after cardiac surgery in the intensive care unit.

## Graphical Abstract

**Figure:**
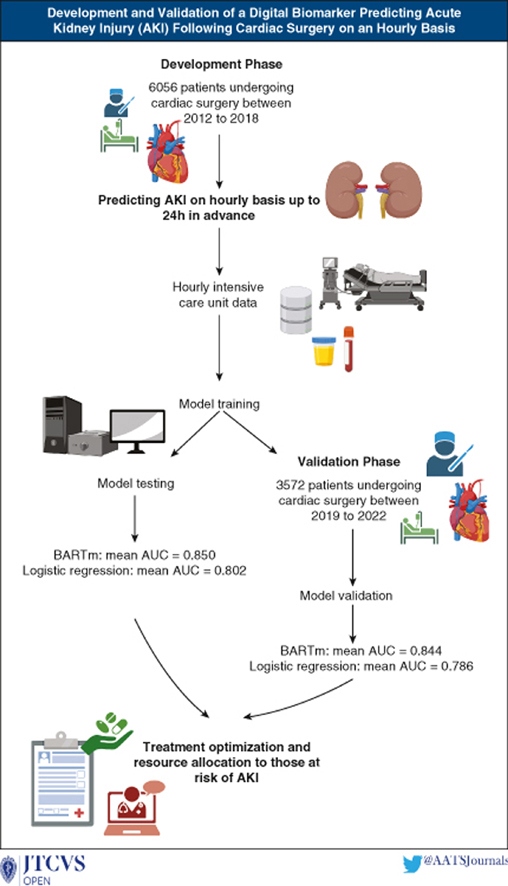

**Figure:**
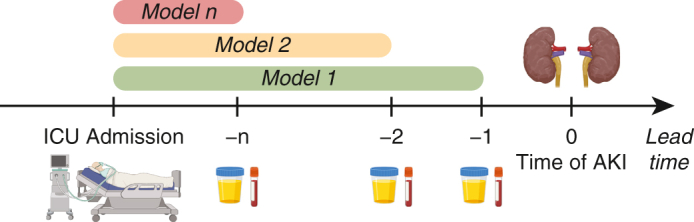
Development of hourly prediction models for acute kidney injury in intensive care.

**Central Message**

Predicting acute kidney injury (AKI) dynamically could help clinicians to optimize treatments and harness preventive strategies for patients at risk of developing AKI after cardiac surgery in the ICU.

**Perspective**

Acute kidney injury (AKI) affects up to 40% of cardiac surgery patients, leading to increased risks of infection, longer hospital stays, and lower quality of life. Currently, there is no single biomarker for AKI. With routine clinical data, AKI was predicted (AUC = 0.850) on an hourly basis in the ICU after cardiac surgery, which will help clinicians with treatment optimization and resource allocation.

Following cardiac surgery, up to 40% of patients can develop acute kidney injury (AKI),^1^ which can contribute to a greater risk of postoperative infection, atrial fibrillation, and a more prolonged stay in the intensive care unit (ICU) and hospital.^2^ Furthermore, AKI is associated with the progression of chronic kidney disease, which affects patients’ long-term quality of life.^3^

Because AKI is a complex, multifactorial complication, there is currently no single molecular or digital biomarker signature that is a so-called “kidney troponin.”^4^ At present, the most promising molecular biomarkers for AKI diagnosis are neutrophil gelatinase-associated lipocalin, interleukin-18, kidney injury molecule-1, cell-cycle arrest biomarkers,^2^ and N-terminal prohormone of brain natriuretic peptide, high-sensitivity C-reactive protein, hemoglobin, and magnesium.^5^ A widely used clinical test for AKI is NEPHROCHECK (NC; Astute Medical), which detects urinary biomarkers tissue inhibitor of metalloproteinases and insulin-like growth-factor binding protein 7 to assess for risk of moderate or severe AKI.^6^ However, these molecular biomarkers are expensive due to requiring extra resources to gather, test, and interpret the data, which consequently affects the usability of these biomarkers.^7^ Therefore, investigating already routinely collected serum data from the ICU to develop a digital biomarker would offer an affordable and automated way to assess the risk of developing AKI.

Within the past decade, numerous dynamic predictive models have been developed with the hope to improve surgical outcomes and overall patient care, mostly to predict mortality and sepsis.^8^ As AKI is a persistent and widespread problem in cardiac surgery, numerous prediction models for AKI have been developed for preoperative use to minimize patient risk before surgery.^2^ However, these models mostly use demographic data, which offer very little granularity when it comes to personalized prediction. Since AKI is still underdiagnosed, especially at lower stages,^9^ having a dynamic, near real-time prediction model suitable for ICU use that considers the patient's physiological changes could be useful to detect AKI hours in advance. A model is considered as dynamic if a prediction is made repeatedly as time and potentially the value associated with each of the predictive variable changes. Using patient data, collected with medical devices and stored in electronic health records, enables the development of a digital biomarker that could be used as a monitoring biomarker^10^ that assesses the status of AKI.

Therefore, with the objective to improve risk assessment for AKI in the ICU for the cardiac population, this study aims to develop and internally validate a digital biomarker to predict the onset of AKI on an hourly basis within 25 hours since ICU admission, up to 24 hours in advance, using routinely collected clinical data.

## Methods

This study gained ethical approval from the responsible UK Health Research Authority (REC18/YH/0366, September 21, 2018). Since this is a retrospective analysis of routinely collected clinical data, the requirement for written informed consent was waived by the Institutional Review Board. This article adheres to the Transparent Reporting of a multivariable prediction model for Individual Prognosis Or Diagnosis guidelines.^11^ The methods used in this study have been described in detail in Appendix E1 (Table E1).

### Predicted Outcome

The Kidney Disease Improving Global Outcomes clinical practice guideline^12^ was used to define AKI. Retrospective diagnosis was given, by dividing each serum creatinine level, measured in the ICU, by the preoperatively measured serum creatinine level (baseline). If the difference was greater than or equal to 1.5 times the baseline, the patient was diagnosed to have AKI. In addition, the timestamp when the creatinine difference occurred was recorded as a timestamp to indicate the occurrence of AKI.

### Setting and Datasets

**Figure 1:**
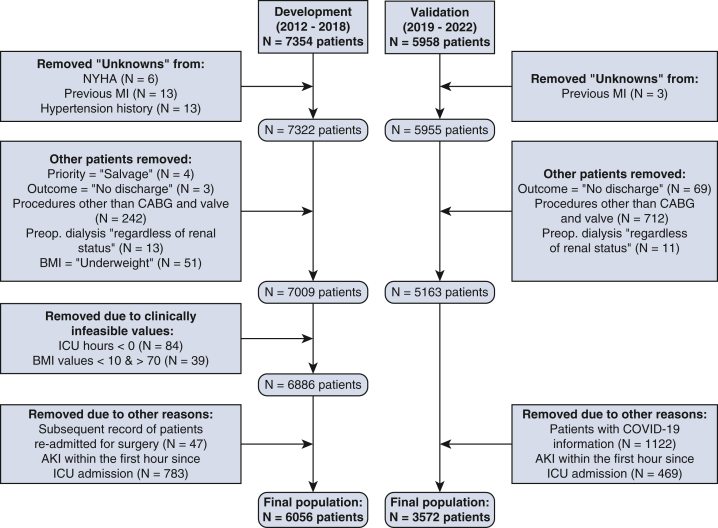
Flow chart of how final patient population was arrived. *NYHA*, New York Heart Association; *MI*, myocardial infarction; *CABG*, coronary artery bypass grafting; *BMI*, body mass index; *ICU*, intensive care unit; *AKI*, acute kidney injury; *COVID-19*, coronavirus disease 2019.

This study was conducted at the Golden Jubilee National Hospital, a large cardiac center in the United Kingdom that performs more than 50% of all elective cardiothoracic surgeries for the National Health Service in Scotland.^13^ Data from 2 local electronic health record databases were used: the Cardiac, Cardiology and Thoracic Health Information database, which includes static information recorded preoperatively, and the Centricity CIS Critical Care database, which includes dynamic laboratory data from the ICU. Data for patients undergoing coronary artery bypass graft (CABG), aortic valve, and combined CABG and valve surgeries between April 1, 2012, and December 31, 2018, were included for the development phase (training and testing) of the models. The patient data between January 1, 2019, and June 30, 2022, was used to internally validate the models. The final number of patients included in this study was 6056 patients for development and 3572 patients for validation. The details of how the final study population for development and validation phase of the study was arrived at are shown in Figure 1.

### Predictors

In total, 82 variables were used in the models, including 25 preoperatively recorded variables, including demographic variables (eg, sex and age), information about the surgery (eg, type and urgency of the surgery), and comorbidities relevant to cardiac surgery (eg, cardiac and renal function). From the ICU database, 13 laboratory variables and 4 medicine-related variables were included. The full list of variables included in the models, together with descriptive statistics can be found from Table E2.

### Classification Methods and Experiments

**Figure 2:**
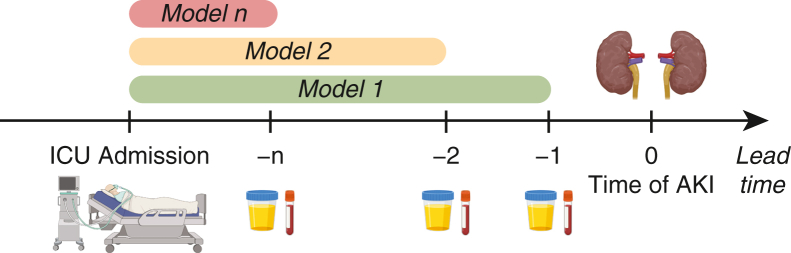
Visualization of how models were developed for each lead time before the event of AKI. *ICU*, Intensive care unit; *AKI*, acute kidney injury.

This paper presents a logistic regression (LR) and a bootstrap aggregated regression trees machine (BARTm) model predicting the onset of AKI within 25 hours since ICU admission on an hourly basis, up to 24 hours in advance. (As part of this study, other methods were also experimented with, the details and results of which can be found from https://stax.strath.ac.uk/concern/theses/6969z130f.) These models were developed for hourly lead times, based on the time windows (Figure 2).

The models were developed on a complete set of training data (ie, all records including missing values were removed). To take advantage of being able to incorporate missing values into the prediction model, 2 experiments were undertaken in terms of incorporating missing values to testing and validation sets.

Experiment 1: Testing and validating the models using complete data (ie, removing all records that included missing values). The results of LR and BARTm are presented.

Experiment 2: Testing and validating the models on datasets that included some missing values. Records with >40% of missing values were excluded from analysis, as done elsewhere.^14^ The rest of the missing values were left as is. Here, the results of BARTm are presented since this method is robust to handle missing data.^15^ The models were developed on the training data, using 10-fold cross validation. All analyses were conducted, using R, version 4.2.2 (R Foundation for Statistical Computing).

The models were evaluated for each lead time using the area under the receiver operating characteristic curve (AUC), sensitivity, specificity, and positive and negative predictive values. The models’ performance measures across all lead times were compared using *t* tests with the significance level set to .05. Also, calibration was assessed through plotting the predicted versus observed probabilities for AKI.

## Results

### Patient Population and AKI

As shown in Table 1, of the 6056 patients included in the development phase, 512 (8.45%) had AKI. Of these patients, 4058 were included in the training set, where the mean age for the training dataset population was 66.08 years, the majority being male (73.04%). The most common procedure was CABG (57.96%). The mean hospital stay was 10.97 days, and the mean ICU stay was 38.94 hours. Overall, 49.41% of the patients had complications and 0.62% of the patients died in the hospital. The testing set of 1998 patients did not significantly differ from the training set population.

**Table 1:** Descriptive statistics of demographic, surgery, and outcome variables based on training, testing (development phase), and validation phase

Variable	Levels	Development phase		*P* value	Validation phase	*P* value
		Train	Test	Test vs train	Validation	Validation vs train
Demographics						
Age	Mean (SD)	66.08 (10.97)	66.26 (10.81)	.5392	65.47 (10.47)	.0171
Sex	Male	2964 (73.04)	1428 (71.47)	.2092	2750 (76.99)	<.0001
	Female	1094 (26.96)	570 (28.53)		822 (23.01)	
Smoking status	Never smoked	1172 (28.88)	559 (27.98)	.1058	1592 (44.57)	<.0001
	Ex-smoker	1253 (30.88)	676 (33.83)		1392 (38.97)	
	Current smoker	561 (13.82)	275 (13.76)		588 (16.46)	
	Unknown	1072 (26.42)	488 (24.42)		0 (0.00)	
BMI	18.5-25.0	750 (18.48)	382 (19.12)	.7926	673 (18.84)	.5644
	25.1-30.0	1607 (39.60)	777 (38.89)		1429 (40.48)	
	>30.0	1701 (41.92)	839 (41.99)		1470 (41.64)	
Surgery						
Surgical priority	Elective	2573 (63.41)	1317 (65.92)	.1721	1146 (32.08)	<.0001
	Emergency	37 (0.91)	13 (0.65)		22 (0.62)	
	Priority	708 (17.45)	314 (15.72)		1198 (33.54)	
	Urgent	740 (18.24)	354 (17.72)		1206 (33.76)	
Surgical procedure	CABG	2352 (57.96)	1159 (58.01)	.0520	2061 (57.70)	<.0001
	Valve	1145 (28.22)	603 (30.18)		852 (23.85)	
	Valve and CABG	561 (13.82)	236 (11.81)		659 (18.45)	
Outcomes						
Outcome	Alive	4033 (99.38)	1982 (99.20)	.5108	3503 (98.07)	<.0001
	Dead	25 (0.62)	16 (0.80)		69 (1.93)	
ICU, h	Mean (SD)	38.94 (68.66)	39.40 (74.09)	.8118	48.69 (104.74)	<.0001
Total days in hospital	Mean (SD)	10.97 (8.37)	10.49 (6.69)	.0248	12.00 (14.31)	<.0001
Acute kidney injury	No	3712 (91.47)	1832 (91.69)	.8121	2977 (83.34)	<.0001
	Yes	346 (8.53)	166 (8.31)		595 (16.66)	

*SD*, Standard deviation; *BMI*, body mass index; *CABG*, coronary artery bypass grafting; *ICU*, intensive care unit.

Of the 3572 patients included in the validation dataset, 595 (16.66%) had AKI. The patients were slightly younger (mean age of 65.47 years), and the proportion of male patients was significantly greater (76.99%) in the validation dataset as compared with the training dataset. The CABG surgery was still the most popular open-heart surgery (57.70%). Hospital stay and ICU hours were significantly different from the training set, with mean ICU hours being 48.69 and total days in hospital being 12.00 days in the validation dataset. A significantly greater proportion of patients in the validation set had complications (62.74%) and passed away (1.93%), compared with the training set.

**Figure E1:**
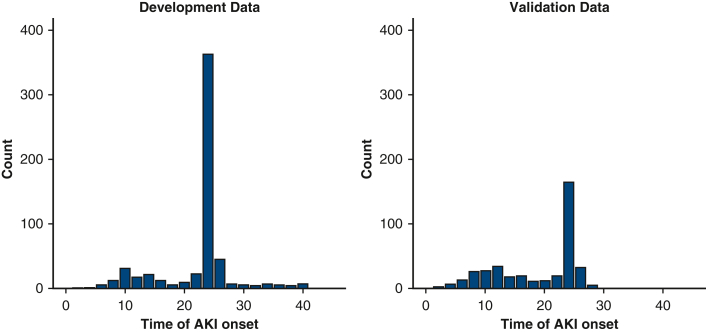
Distribution of how time of acute kidney injury is diagnosed in the ICU in development phase (training and testing data) and validation datasets. *AKI*, Acute kidney injury; *ICU*, intensive care unit.

Detailed descriptive statistics of all variables and comparison between the training, testing, and validation datasets can be found from Table E2. Most patients in both development and validation datasets had AKI between 20 and 25 hours since ICU admission (Figure E1), more specifically at median hours of 16.18 (interquartile range, 24.49) in development phase data and 19.78 (interquartile range, 23.95) in validation data. Interestingly, patients in the validation data appeared to have the onset of AKI in general earlier than in the development dataset. This is because AKI was retrospectively diagnosed using serum creatinine measurements and, as shown in Table 2, creatinine measurements were taken more frequently in validation dataset than in dataset recorded in the development phase.

**Table 2:** Mean and standard deviation (SD) hours of when each laboratory variable is recorded in development and validation datasets, where *P* value signifies whether there is a statistically significant difference between the frequency of measurement between development phase and validation phase

Variable	Development phase (2012-2018)	Validation phase (2019-2022)	*P* value
	Mean (SD) hours	Mean (SD) hours	
Every 10 h			
Creatinine	10.52 (11.60)	6.36 (10.16)	<.0001
Urea	10.52 (11.60)	6.35 (10.15)	<.0001
C-reactive protein	10.40 (11.73)	6.13 (10.13)	<.0001
Every 1-2 h			
Arterial base excess	1.45 (1.21)	1.08 (1.27)	<.0001
Arterial hematocrit	1.51 (1.30)	1.08 (1.26)	<.0001
Bicarbonate	1.45 (1.22)	1.08 (1.31)	<.0001
Hemoglobin	1.48 (1.25)	1.08 (1.26)	<.0001
Hydrogen ion	1.44 (1.19)	1.08 (1.26)	<.0001
Lactate	2.04 (1.35)	1.09 (1.28)	<.0001
Potassium	1.45 (1.20)	1.08 (1.26)	<.0001
Sodium	1.45 (1.20)	1.08 (1.26)	<.0001
Depending on patient			
Urine output	0.64 (0.57)	0.73 (0.60)	<.0001
Daily fluid balance	7.76 (10.98)	6.98 (10.57)	<.0001

*SD*, Standard deviation.

It is important to note that there appear to be significant differences in the development and validation patient populations (Table 1 and Table E2) in terms of characteristics, but also in terms of frequency of data collection in the ICU (Table 2). The reasons for this are multifactorial and hence difficult to objectively underpin. It can be speculated that the differences could be due to the changing procedures, where more straightforward patients tend to have more minimally invasive surgeries, such as percutaneous coronary intervention, as opposed to riskier CABG and/or valve surgeries.^16^ Changes in patient population can also occur due to policy changes in patient selection processes but also changes in data collection.^17^ However, the frequency of data collection could also be different simply due to improvement and automation of the devices collecting the data.^18^

### Models Predicting Acute Kidney Injury in ICU on Hourly Basis

#### Models’ discrimination

**Figure 3:**
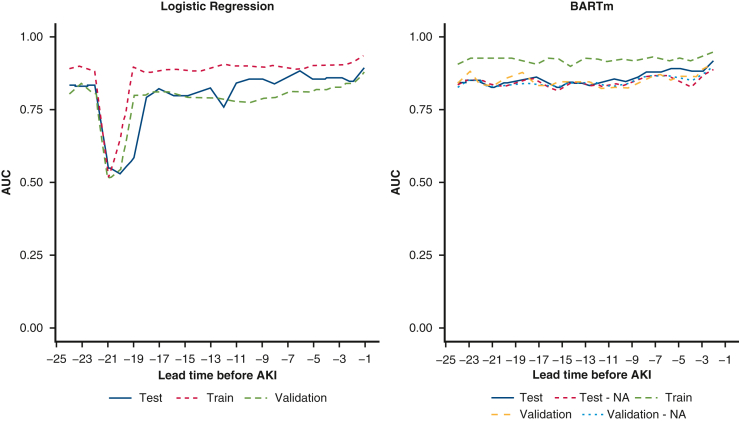
Area under the receiver operating characteristic curve (*AUC*) for both models for each lead time, applied to training, testing and validation datasets. *AKI*, Acute kidney injury; *BARTm*, bootstrap aggregated regression trees machine.

**Figure E2:**
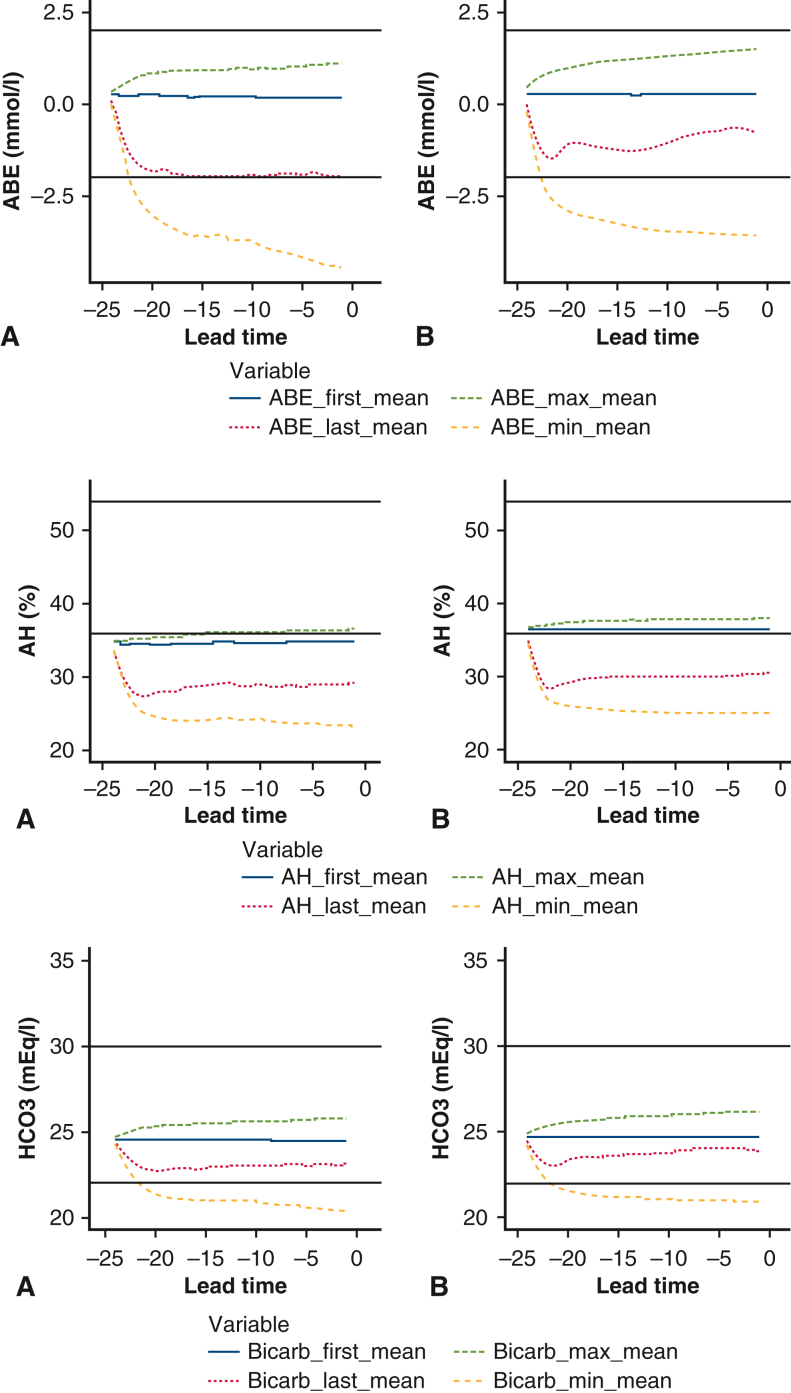

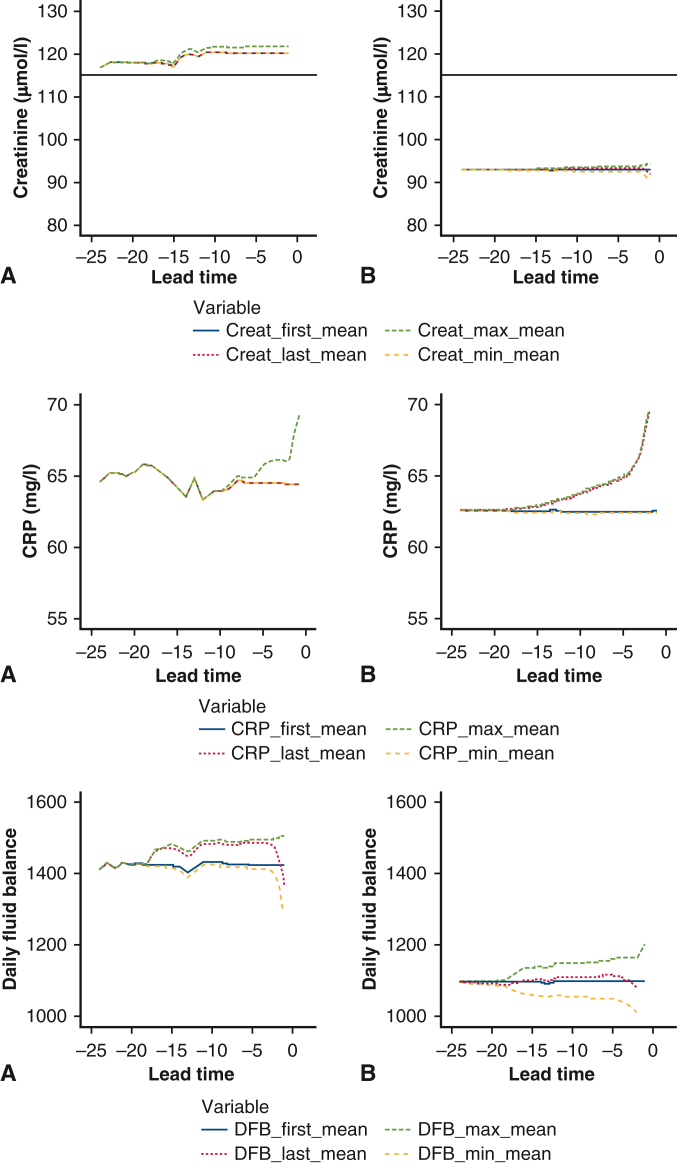

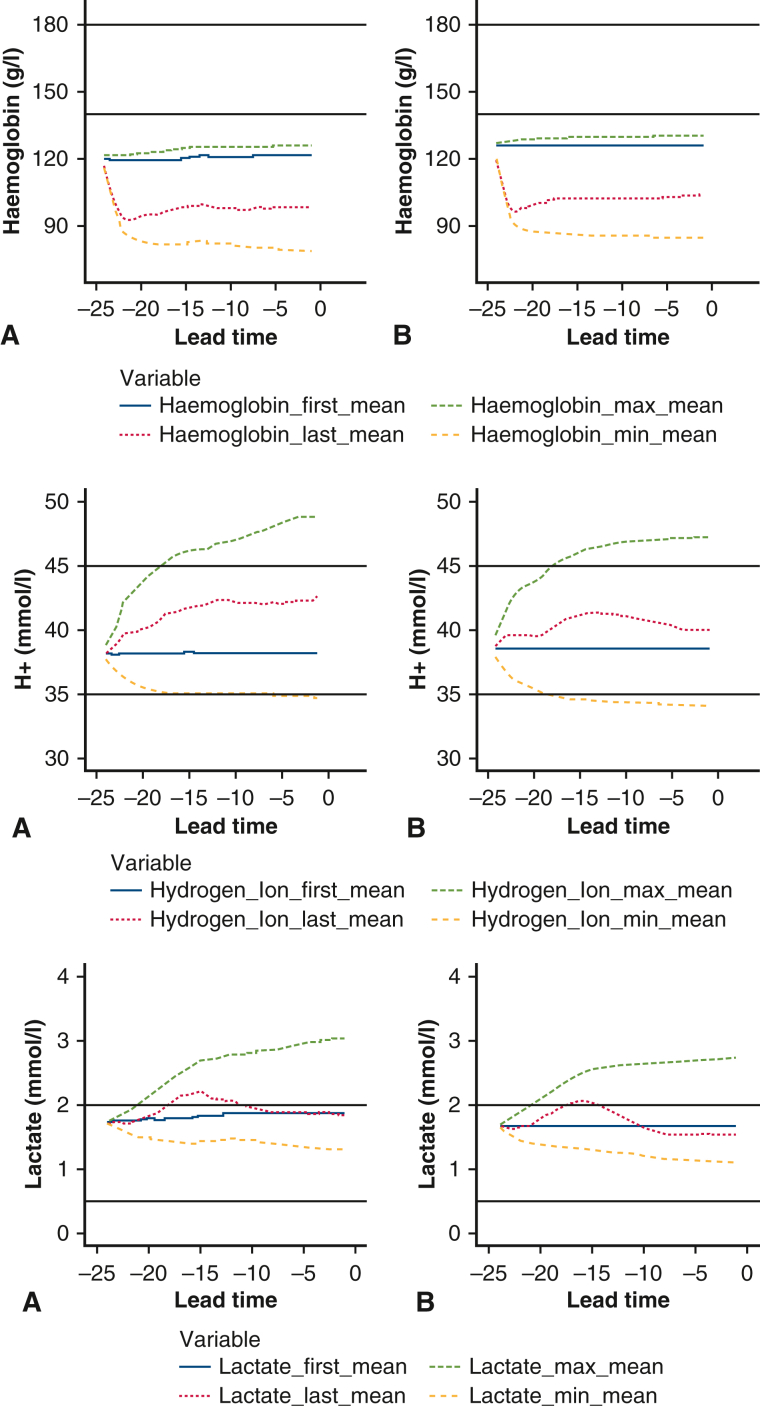

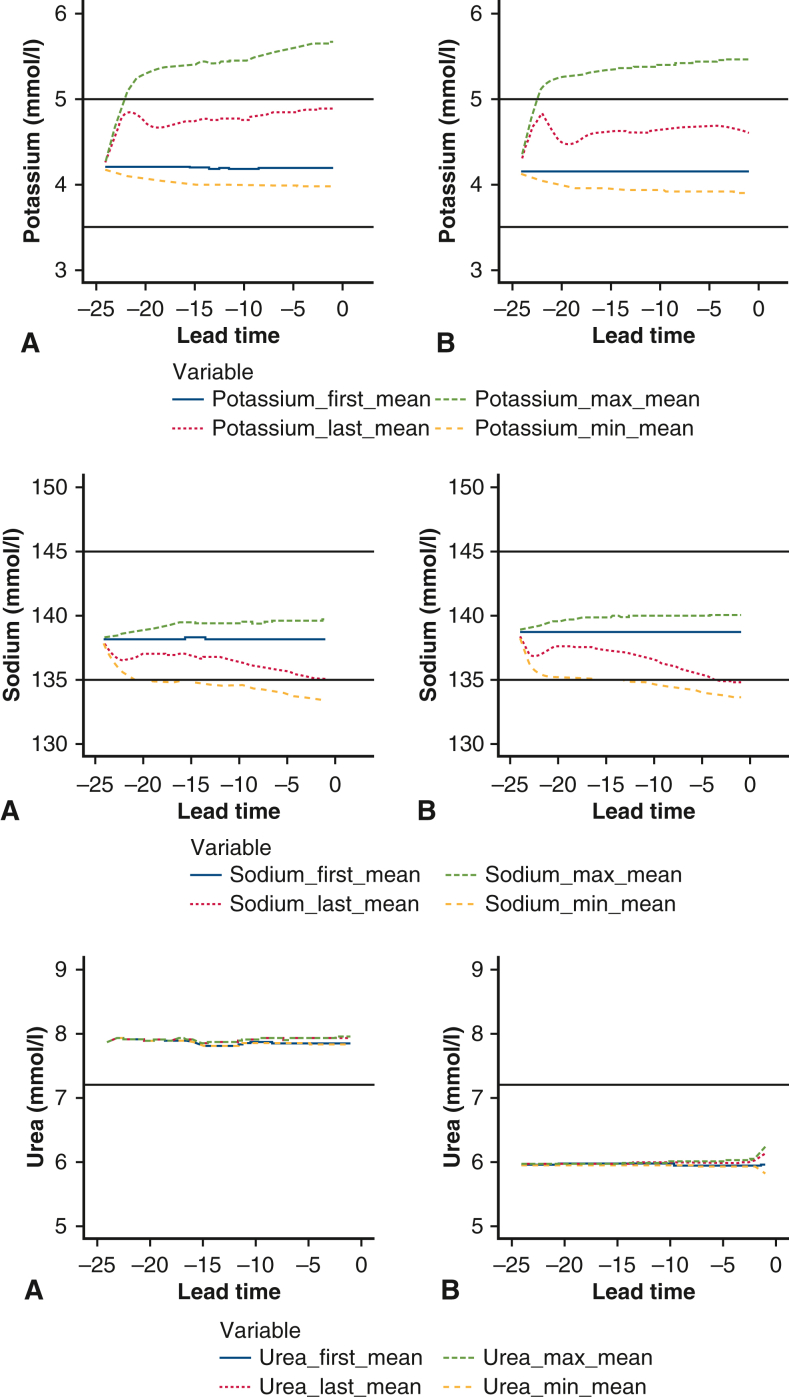

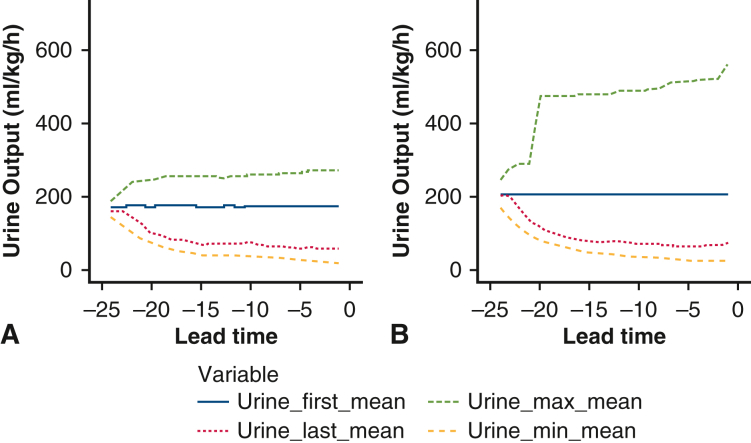
The changing of laboratory variable summary values as the lead time changes for patients with acute kidney injury (AKI) (A) and for patients without AKI (B). The *horizontal lines* indicate the normal range for each retrospective laboratory value. *ABE*, Arterial base excess; *AH*, arterial hematocrit; *HCO3*, bicarbonate; *CRP*, C-reactive protein; *DFB*, daily fluid balance; *H+*, hydrogen ion.

For both models, the performance, regardless of training, testing or validation datasets, tended to increase as the lead time got closer to 0 (Figure 3). The reason behind this might be that with shorter lead times more data were available for each patient, giving the algorithms more information from which to construct a model that could indicate the probability whether the patient would have AKI. However, interestingly, at the lead times 22 and 21, the LR model had a noticeable dip in performance. This could be due to more variation being introduced to the model as more data was entered into the system at these time windows (Figure E2).

The BARTm model using complete training data and complete testing data (Experiment 1) achieved the greatest mean AUC of 0.850 and the greatest mean sensitivity of 0.821 (Table 3) (mean variable importance reported in Table E3). Logistic regression from Experiment 1 had the greatest mean specificity of 0.824 (model coefficients reported in Tables E4 and E5). In terms of negative predictive value, BARTm developed with complete training data and tested with missing values (Experiment 2) achieved a greater negative predictive value of 0.800 than LR. For both models in both experiments, the positive predictive values were very low due to low prevalence of AKI in the patient population. In fact, based on the mean AUC, BARTm had a significantly greater performance than LR, with the mean AUC of 0.923 for training, AUC of 0.850 for testing and 0.844 for validation data.

**Table 3:** Mean and standard deviation (SD) of each performance measure for training, testing, and validation data for both BARTm and LR models

Performance measure	Data	BARTm (mean, SD)	LR (mean, SD)	*P* value (BARTm vs LR)
AUC	Training	0.923 (0.011)	0.872 (0.093)	.0142
	Testing – complete	0.850 (0.026)	0.802 (0.100)	.0324
	Testing – NA	0.837 (0.018)		
	Validation – complete	0.844 (0.024)	0.786 (0.083)	.0026
	Validation – NA	0.838 (0.020)		
Sensitivity	Training	0.875 (0.042)	0.760 (0.189)	.0075
	Testing – complete	0.821 (0.053)	0.668 (0.216)	.0024
	Testing – NA	0.811 (0.050)		
	Validation – complete	0.789 (0.045)	0.667 (0.196)	.0063
	Validation – NA	0.767 (0.048)		
Specificity	Training	0.818 (0.042)	0.844 (0.050)	.0523
	Testing – complete	0.741 (0.057)	0.824 (0.080)	.0002
	Testing – NA	0.716 (0.058)		
	Validation – complete	0.806 (0.062)	0.817 (0.073)	.5770
	Validation – NA	0.774 (0.037)		
PPV	Training	0.021 (0.028)	0.022 (0.012)	.8383
	Testing – complete	0.021 (0.006)	0.038 (0.037)	.0339
	Testing – NA	0.021 (0.005)		
	Validation – complete	0.019 (0.004)	0.019 (0.007)	.6671
	Validation – NA	0.025 (0.034)		
NPV	Training	0.700 (0.044)	0.692 (0.044)	.5438
	Testing – complete	0.775 (0.054)	0.742 (0.076)	.0860
	Testing – NA	0.807 (0.036)		
	Validation – complete	0.758 (0.055)	0.835 (0.044)	<.0001
	Validation – NA	0.823 (0.030)		

Here, “NA” denotes that missing values were included in the dataset, as was done in Experiment 2. *BARTm*, Bootstrap aggregated regression trees machine; *SD*, standard deviation; *LR*, logistic regression; *AUC*, area under the receiver operating characteristic curve; *PPV*, positive predictive value; *NPV*, negative predictive value.

BARTm performed comparably well, when applied to testing and validation datasets that included missing values, with mean AUC being 0.837 and 0.838 for testing and validation datasets, respectively. This result is very promising, because missing data in routinely collected clinical data are common^19^ and being able to apply the model on patients whose data is not complete can be extremely helpful to predict AKI in practice.

**Figure 4:**
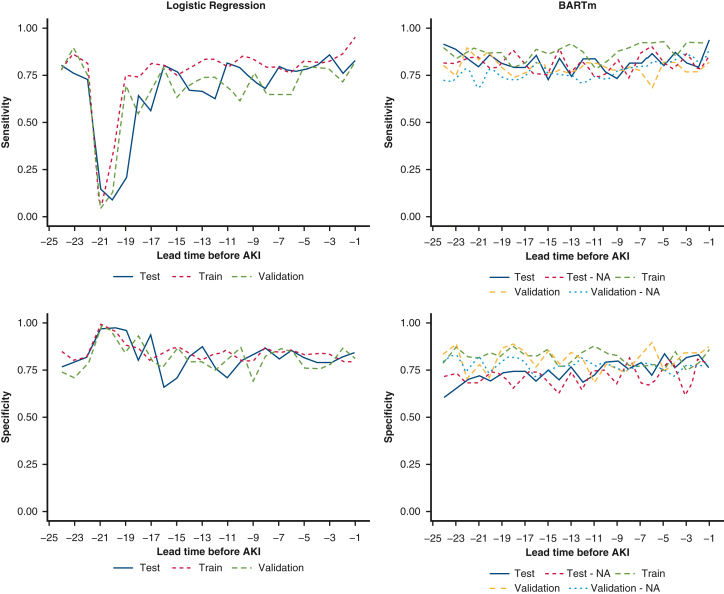
Sensitivity and specificity for both models for each lead time, applied to training, testing and validation datasets. *AKI*, Acute kidney injury; *BARTm*, bootstrap aggregated regression trees machine.

There is a noticeable variation in sensitivity and specificity (Figure 4) from one lead time to another, especially for logistic regression between lead times of −18 and −22, again likely due to introduction of more variation in laboratory values at these lead times. The exact performance measures for each lead time for each model and experiment can be found from Table E6.

#### Calibration of the models

**Figure E3:**
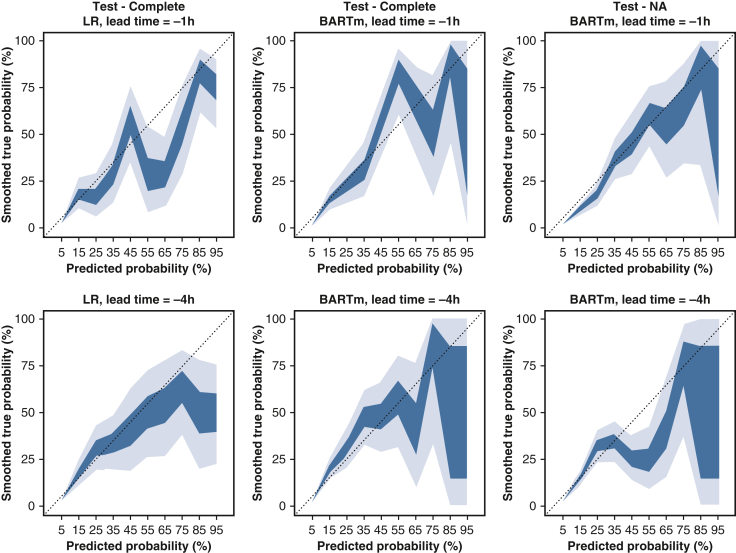

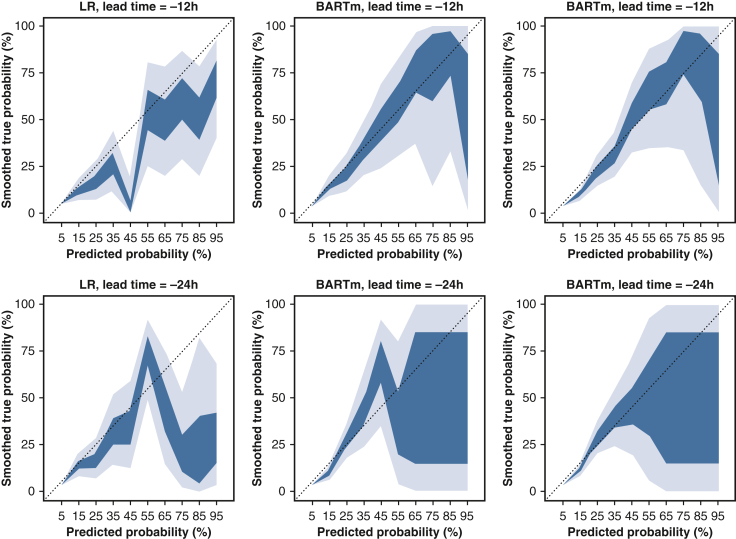
Calibration for logistic regression (*LR*) and bootstrap aggregated regression trees machine (*BARTm*) models, when applied to complete test sets and test sets with missing values (test–NA) at 1, 4, 12, and 24 hours before the onset of AKI. *AKI*, Acute kidney injury.

**Figure E4:**
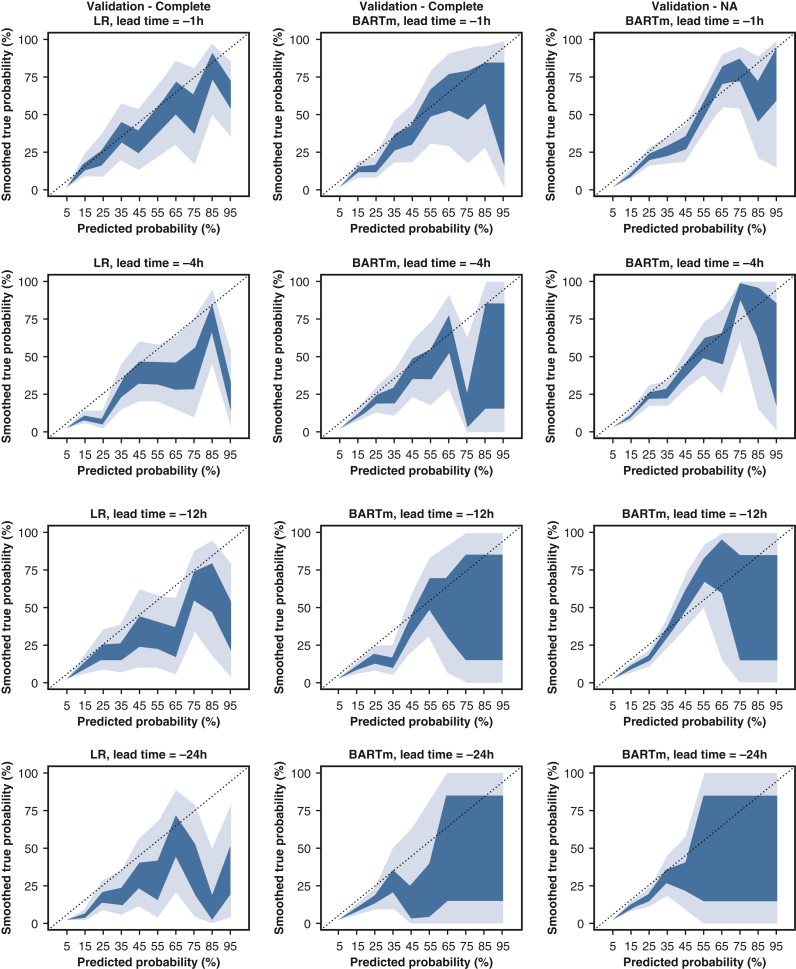
Calibration for logistic regression (*LR*) and bootstrap aggregated regression trees machine (*BARTm*) models, when applied to complete validation sets and validation sets with missing values (validation–NA) at 1, 4, 12, and 24 hours before the onset of AKI. *AKI*, Acute kidney injury.

Unsurprisingly, models were more confident at their predictions at lead times, which were closer to the onset of AKI (ie, at 1 hour and 4 hours in advance) than when the prediction was made earlier (Figures E3 and E4). Furthermore, in all experiments, both models were more confident at predicting patients to not have AKI (ie, when the probability of AKI is low), rather than at predicting patients to have AKI. This is especially evident when looking at the BARTm model predicting AKI 24 hours in advance. The models tend to slightly overestimate the risk of AKI if the actual probability is low, and underestimate if the actual probability is high. The mean predicted probabilities and actual proportion of patients with AKI are shown for each model at each lead time for each experiment in Table E7.

## Discussion

### Summary of Results and Comparison with Existing Models

**Figure 5:**
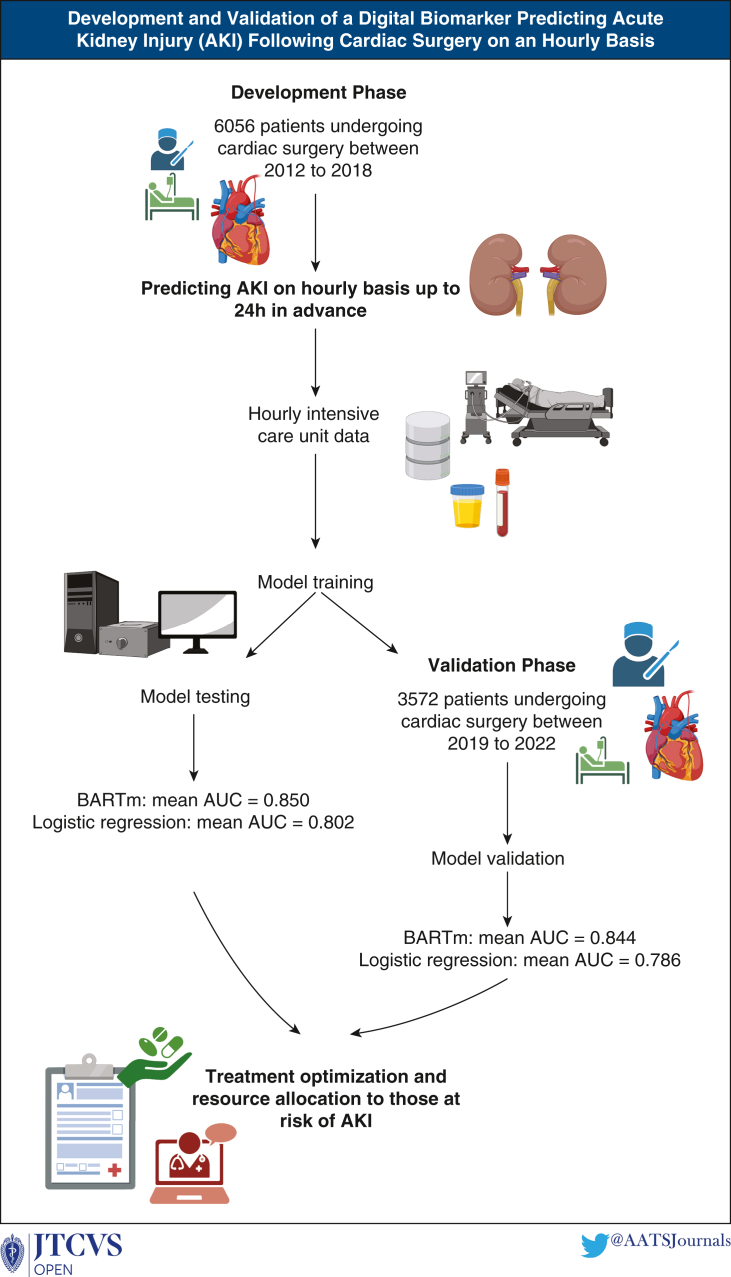
The process of how the digital biomarkers were developed to predict acute kidney injury on an hourly basis. *BARTm*, Bootstrap aggregated regression trees machine; *AUC*, area under the receiver operating characteristic curve.

This study developed and validated a digital biomarker that predicts AKI in ICU following cardiac surgery on an hourly basis (Figure 5). The best-performing model, BARTm achieved high overall performance on testing data (mean AUC = 0.850, sensitivity = 0.821 and specificity = 0.741) and validation data (mean AUC = 0.844, sensitivity = 0.789, and specificity = 0.806). The model also predicted AKI when data included missing values, achieving mean AUC of 0.837 for testing data and 0.838 for validation data. Even though AKI is a persistent and widespread problem in cardiac surgery, only 2 dynamic prediction models for AKI have been developed to date.^20^^,^^21^

Meyer and colleagues^20^ predicting renal failure achieved greater performance (AUC of 0.96, sensitivity of 0.94 and specificity of 0.86), whereas the BARTm model outperformed Ryan and colleagues's^21^ model (AUC = 0.82) when predicting any stage of AKI within 48 hours since ICU admission.

Both Meyer and colleagues'^20^ and Ryan and colleagues'^21^ models have some limitations, such as potential overestimation of predicted outcome due to balancing methods,^22^ which could lead to poor calibration.^23^ Neither of the studies report their models' calibration, making it difficult to compare these models' applicability with the model developed in this study in clinical practice.

When we compared the digital biomarker developed as part of this study with the widely used NC urine biomarker test, BARTm noticeably achieved a better AUC, sensitivity, and specificity than the NC (AUC = 0.633, sensitivity = 0.56, and specificity = 0.64), achieved by the development study.^6^ Although the NC has shown to have a great performance when applied to patients who undergo cardiac surgery (AUC = 0.84, sensitivity = 0.92, and specificity = 0.81),^24^ the performance of the NC has not been consistent, only achieving an AUC of 0.60 in a recent study investigating off-pump CABG patients.^25^ It is important to note that due to the nature of the cost of testing molecular biomarkers, these studies validating NC are very small, including only 50 and 90 patients, respectively.

### Strengths and Limitations

Although the Kidney Disease Improving Global Outcomes criteria are currently the most objective and accurate way to diagnose AKI,^2^ they rely on serum creatinine laboratory results. Since creatinine was measured more frequently in validation phase than in development phase (Table 2), the hourly prediction based on more frequent creatinine measurements could improve diagnosis reliability, which could be an explanation for why the models still performed well in the validation datasets, regardless of the validation and the development phase data being significantly different based on the frequency of measurements and also values. Since this study is a single-center study, it is unclear whether creatinine is measured more frequently in the later years as an international standard, or whether this change took place simply at the study institution. Therefore, it is unclear whether the models could perform well in validation data where the creatinine measurements are either the same as in the development phase or even less frequent. To answer this question, an external validation study is needed.

Due to the missing values of hemoglobin in earlier years in the Cardiac, Cardiology and Thoracic Health Information database, preoperatively measured hemoglobin variable was excluded from the analysis. As hemoglobin has been shown to be associated with kidney function, the exclusion of this variable can be perceived as a limitation of this study. However, as the models presented in this study integrate the latest laboratory information available on an hourly basis, the significance of the most recent hemoglobin level, documented within the ICU, outweighs the importance of the hemoglobin level recorded during the pre-operative phase at the clinic. In the ICU, hemoglobin was recorded every 1 to 1.5 hours (Table 2) for 99.9% to 100% of patients (Table E2), making it a more reliable measure than preoperative hemoglobin. Although we have made use of BARTm's capability to consider incomplete data for ICU laboratory measurements, we have opted not to apply data imputation methods to address missing values in the preoperative hemoglobin measurements. This decision is based on the availability of more dependable and current hemoglobin data within the ICU, and our desire to prevent potential biases that imputation methods might introduce.^26^

Missing data in electronic health records are very common and are a barrier to development of accurate and usable clinical prediction models.^19^ The competitive performance by BARTm with missing values on testing (mean AUC = 0.830) and validation data (mean AUC = 0.838) is promising. Being able to use methods that can make a prediction, even with the presence of missing data, can be extremely beneficial as a clinician can still be informed whether a patient is likely to develop AKI due to the well-performing model that is robust to missing values. In the future, the models should also be tested on datasets including larger proportions of missing data as entries with more than 40% of missing values were removed from analysis.

The reduced interpretability of BARTm compared with logistic regression poses a challenge due to the lack of model coefficients. However, since ICU is a complex, data-rich environment, to put either of these models into use in practice, clinical software needs to be developed to apply the models to patient data.

Finally, using a local dataset may limit generalizability but ensures greater relevance of the models within this specific setting. Local care processes can vary between institutions, and policies influencing treatment and access to care can differ across countries, and therefore, external validation and recalibration are needed to support applicability to other populations.^27^

### Clinical Implications and Future Work

The hourly ICU digital biomarker has the potential to be developed into a clinical system that is integrated with electronic health records. Such a system could aid clinicians in risk assessment, treatment planning, and resource allocation to predict AKI hours in advance. The work presented in this article is the first step to developing the clinical decision support model that is integrated with the electronic health records in the ICU, as is done with the current commonly used risk prediction models. Unlike the Sequential Organ Failure Assessment and Acute Physiology, Age and Chronic Health Evaluation scores,^28^ the digital biomarker calculates the risk every hour, allowing clinicians to find out which patients are at risk of developing AKI in a timely manner, well in advance to avoid late diagnosis, and consequently worsened health outcomes for patients.

As AKI is still vastly underdiagnosed,^9^ there is a need for an accurate, usable, and timely way to diagnose AKI, for which the BARTm is a great candidate. The high sensitivity and specificity show the model's ability to recognize patients with and without AKI comparatively well. The negative predictive value staying above 0.700 for development, testing, and validation sets shows the model classifies patients to be without AKI with a 70% probability. Although there is room for improvement regarding false positives and false negatives, it is unknown whether this model performs better than other models in that regard as the other similar studies have not reported this information.^20^^,^^21^

To improve the predictive ability of the models, in the future, the inclusion of vital signs, molecular serum, and plasma data could be beneficial.^2^ Furthermore, to improve the usability and applicability of the models, other complications that are known to be associated with AKI, such as delirium and sepsis, could be added as additional outcomes to be predicted. Although the data from the validation phase were significantly different from the development phase, interestingly, the models performed well at predicting AKI on the validation set, based on discrimination, and calibration. As mentioned earlier, although the reasons for the development and validation datasets being different are multifactorial and therefore difficult to objectively underpin, the strong performance of the models in the validation set shows the robustness of the models to the possible changes in patient population, health policies, and changes in medical devices, ICU protocols, patient pathways, and even to possible effects on changes in patient selection due to the coronavirus disease 2019 pandemic. However, to confirm the robustness of the model and to support its generalizability before implementation into clinical practice,^27^ an external validation study, an updating strategy, and a clinical support system integrated with electronic health records are needed for widespread adoption.

In summary, this study developed a digital biomarker for hourly prediction of AKI in the ICU after cardiac surgery, demonstrating high performance. These digital biomarkers could help clinicians optimize treatments for patients who are at risk of developing AKI hours in advance.

## Conflict of Interest Statement

The authors reported no conflicts of interest.

The *Journal* policy requires editors and reviewers to disclose conflicts of interest and to decline handling or reviewing manuscripts for which they may have a conflict of interest. The editors and reviewers of this article have no conflicts of interest.

## Figures and Tables

**Table E1:** Number of patients in training, testing, and validation datasets, with proportion of patients with AKI and missingness of the datasets

Lead time	Training data (100% complete)		Testing data			Validation data		
	Number of patients	AKI (%)	Number of patients	Missingness (%)	AKI (%)	Number of patients	Missingness (%)	AKI (%)
Experiment 1								
−24	2417	6.33	1189	0.00	6.81	1276	0.00	3.53
−23	2427	6.88	1195	0.00	6.61	1285	0.00	3.81
−22	2431	7.03	1197	0.00	6.77	1297	0.00	3.85
−21	2433	6.86	1197	0.00	7.27	1306	0.00	3.98
−20	2437	7.18	1199	0.00	7.01	1309	0.00	4.05
−19	2438	6.97	1200	0.00	7.58	1314	0.00	4.11
−18	2439	7.18	1201	0.00	7.33	1315	0.00	4.18
−17	2443	7.29	1202	0.00	7.49	1316	0.00	4.25
−16	2445	7.69	1203	0.00	6.90	1321	0.00	4.54
−15	2450	7.55	1206	0.00	7.79	1323	0.00	4.69
−14	2456	7.9	1209	0.00	7.78	1324	0.00	4.76
−13	2462	7.88	1212	0.00	8.33	1328	0.00	5.04
−12	2468	8.14	1215	0.00	8.31	1330	0.00	5.11
−11	2475	8.36	1218	0.00	8.54	1333	0.00	5.18
−10	2482	8.78	1221	0.00	8.44	1337	0.00	5.38
−9	2485	9.01	1223	0.00	8.18	1341	0.00	5.52
−8	2488	8.68	1224	0.00	9.15	1344	0.00	5.51
−7	2492	8.87	1226	0.00	9.05	1349	0.00	5.63
−6	2492	8.87	1226	0.00	9.05	1350	0.00	5.70
−5	2492	8.79	1227	0.00	9.29	1350	0.00	5.70
−4	2492	8.79	1227	0.00	9.29	1350	0.00	5.70
−3	2492	8.79	1227	0.00	9.29	1350	0.00	5.70
−2	2493	8.78	1227	0.00	9.37	1351	0.00	5.77
−1	2495	9.26	1228	0.00	8.39	1351	0.00	5.77
Mean (SD)	2464 (25.75)	7.99 (0.85)	1212 (2.69)		8.08 (0.91)	1327 (21.78)		4.89 (0.76)
Experiment 2								
−24	2417	6.33	1722	3.12	6.39	2299	3.58	4.22
−23	2427	6.88	1727	3.10	6.43	2307	3.51	4.55
−22	2431	7.03	1728	3.09	6.48	2309	3.45	4.63
−21	2433	6.86	1729	3.09	6.88	2313	3.41	4.80
−20	2437	7.18	1729	3.07	6.65	2315	3.40	4.88
−19	2438	6.97	1729	3.07	7.00	2316	3.39	4.92
−18	2439	7.18	1732	3.07	6.93	2320	3.39	5.09
−17	2443	7.29	1734	3.07	7.09	2322	3.39	5.17
−16	2445	7.69	1735	3.07	6.69	2328	3.39	5.41
−15	2450	7.55	1738	3.06	7.31	2331	3.39	5.53
−14	2456	7.9	1741	3.06	7.29	2332	3.38	5.57
−13	2462	7.88	1742	3.05	7.69	2340	3.38	5.90
−12	2468	8.14	1743	3.04	7.69	2345	3.39	6.10
−11	2475	8.36	1745	3.03	7.85	2349	3.38	6.26
−10	2482	8.78	1749	3.03	7.83	2356	3.37	6.54
−9	2485	9.01	1749	3.02	7.66	2362	3.37	6.77
−8	2488	8.68	1750	3.02	8.34	2365	3.36	6.89
−7	2492	8.87	1750	3.01	8.29	2367	3.34	6.97
−6	2492	8.87	1750	3.00	8.29	2369	3.33	7.05
−5	2492	8.79	1751	3.00	8.45	2370	3.34	7.09
−4	2492	8.79	1751	3.00	8.45	2370	3.34	7.09
−3	2492	8.79	1751	3.00	8.45	2371	3.34	7.13
−2	2493	8.78	1751	2.99	8.51	2373	3.34	7.21
−1	2495	9.26	1749	2.97	7.83	2373	3.34	7.21
Mean (SD)	2464 (25.75)	7.99 (0.85)	1740 (9.75)	3.04 (0.04)	7.52 (0.73)	2341 (25.18)	3.39 (0.06)	5.96 (1.01)

*AKI*, Acute kidney injury; *SD*, standard deviation.

**Table E2:** Descriptive statistics of all variables included in the models, where frequencies and percentages are shown for categorical and mean and standard deviation (SD) are shown for numerical variables

Variable	Levels	Development data			*P* value	Validation data		*P* value
		Completeness (%)	Train	Test	Test vs train	Completeness (%)	Validation	Validation vs train
Demographics								
Age	Mean (SD)	100.0	66.08 (10.97)	66.26 (10.81)	.5392	100.0	65.47 (10.47)	.0171
Sex	Male	100.0	2964 (73.04)	1428 (71.47)	.2092	100.0	2750 (76.99)	<.0001
	Female		1094 (26.96)	570 (28.53)			822 (23.01)	
Smoking status	Never smoked	74.2	1172 (28.88)	559 (27.98)	.1058	100.0	1592 (44.57)	<.0001
	Ex-smoker		1253 (30.88)	676 (33.83)			1392 (38.97)	
	Current smoker		561 (13.82)	275 (13.76)			588 (16.46)	
	Unknown		1072 (26.42)	488 (24.42)			0 (0.00)	
BMI	18.5-25.0	100.0	750 (18.48)	382 (19.12)	.7926	100.0	673 (18.84)	.5644
	25.1-30.0		1607 (39.60)	777 (38.89)			1429 (40.48)	
	>30.0		1701 (41.92)	839 (41.99)			1470 (41.64)	
Comorbidities								
Logistic EuroSCORE	Mean (SD)	100.0	4.96 (5.52)	5.02 (5.51)	.7046	100.0	5.41 (7.13)	.0014
Diabetes	No	100.0	3051 (75.18)	1496 (74.87)	.8176	100.0	2542 (71.16)	.0005
	Yes		1007 (24.82)	502 (25.13)			1030 (28.84)	
Renal impairment	Normal	76.4	1690 (41.65)	811 (40.59)	.5067	100.0	2084 (58.34)	<.0001
	Moderate		1129 (27.82)	585 (29.28)			1261 (35.30)	
	Severe		268 (6.60)	142 (7.11)			227 (6.35)	
	Unknown		971 (23.93)	460 (23.02)			0 (0.00)	
Neurologic dysfunction	No	100.0	4013 (98.89)	1967 (98.45)	.1828	100.0	3244 (90.82)	<.0001
	Yes		45 (1.11)	31 (1.55)			328 (9.18)	
Preoperative creatinine	Mean (SD)	100.0	89.82 (42.39)	92.25 (62.43)	.0743	100.0	89.85 (34.63)	.4295
Pulmonary disease	No	100.0	3380 (83.29)	1691 (84.63)	.1957	100.0	3003 (84.07)	.6717
	Yes		678 (16.71)	307 (15.37)			569 (15.93)	
Cardiac health								
LV function	Good	100.0	3222 (79.40)	1612 (80.68)	.0907	100.0	2655 (74.33)	<.0001
	Moderate		712 (17.55)	344 (17.22)			805 (22.54)	
	Poor		124 (3.06)	42 (2.10)			112 (3.14)	
NYHA grade	I	100.0	844 (20.80)	389 (19.47)	.0805	100.0	732 (20.49)	<.0001
	II		2052 (50.57)	1001 (50.10)			1592 (44.57)	
	III		1027 (25.31)	556 (27.83)			1099 (30.77)	
	IV		135 (3.33)	52 (2.60)			149 (4.17)	
Angina status	0	100.0	1278 (31.49)	641 (32.08)	.6557	100.0	1082 (30.29)	<.0001
	I		546 (13.45)	279 (13.96)			698 (19.54)	
	II		1376 (33.91)	688 (34.43)			1059 (29.65)	
	III		645 (15.89)	298 (14.91)			576 (16.13)	
	IV		213 (5.25)	92 (4.60)			157 (4.40)	
Rhythm	Normal	94.2	3371 (83.07)	1657 (82.93)	.1860	99.4	3161 (88.49)	<.0001
	Abnormal		438 (10.79)	237 (11.86)			388 (10.86)	
	Unknown		249 (6.14)	104 (5.21)			23 (0.64)	
Previous MI	No	100.0	2548 (62.79)	1242 (62.16)	.6555	100.0	2105 (58.93)	.0015
	Yes		1510 (37.21)	756 (37.84)			1467 (41.07)	
LMS disease	No	61.1	1879 (46.30)	972 (48.65)	.1250	93.8	2750 (76.99)	<.0001
	Yes		565 (13.92)	285 (14.26)			600 (16.80)	
	Unknown		1614 (39.77)	741 (37.09)			222 (6.22)	
Hypertension history	No	100.0	1122 (27.65)	567 (28.38)	.5724	100.0	1021 (28.58)	.3531
	Yes		2936 (72.35)	1431 (71.62)			2551 (71.42)	
Congestive cardiac failure	No	100.0	3685 (90.81)	1809 (90.54)	.7714	100.0	2989 (83.68)	<.0001
	Yes		373 (9.19)	189 (9.46)			583 (16.32)	
Previous operations	No	100.0	3969 (97.81)	1948 (97.50)	.5064	100.0	3433 (96.11)	<.0001
	Yes		89 (2.19)	50 (2.50)			139 (3.89)	
Previous PCI	No	100.0	3485 (85.88)	1753 (87.74)	.0513	100.0	2940 (82.31)	<.0001
	Yes		573 (14.12)	245 (12.26)			632 (17.69)	
Active endocarditis	No	100.0	4019 (99.04)	1979 (99.05)	1.0000	100.0	3501 (98.01)	.0001
	Yes		39 (0.96)	19 (0.95)			71 (1.99)	
Surgery								
Surgical priority	Elective	100.0	2573 (63.41)	1317 (65.92)	.1721	100.0	1146 (32.08)	<.0001
	Emergency		37 (0.91)	13 (0.65)			22 (0.62)	
	Priority		708 (17.45)	314 (15.72)			1198 (33.54)	
	Urgent		740 (18.24)	354 (17.72)			1206 (33.76)	
Surgical procedure	CABG	100.0	2352 (57.96)	1159 (58.01)	.0520	100.0	2061 (57.70)	<.0001
	Valve		1145 (28.22)	603 (30.18)			852 (23.85)	
	Valve and CABG		561 (13.82)	236 (11.81)			659 (18.45)	
Extracardiac arteriopathy	No	100.0	3615 (89.08)	1757 (87.94)	.2003	100.0	3215 (90.01)	.0128
	Yes		443 (10.92)	241 (12.06)			357 (9.99)	
Critical preoperative state	No	100.0	3994 (98.42)	1966 (98.40)	1.0000	100.0	3469 (97.12)	.0001
	Yes		64 (1.58)	32 (1.60)			103 (2.88)	
Outcomes								
Outcome	Alive	100.0	4033 (99.38)	1982 (99.20)	.5108	100.0	3503 (98.07)	<.0001
	Dead		25 (0.62)	16 (0.80)			69 (1.93)	
ICU hours	Mean (SD)	100.0	38.94 (68.66)	39.40 (74.09)	.8118	100.0	48.69 (104.74)	<.0001
Total days in hospital	Mean (SD)	100.0	10.97 (8.37)	10.49 (6.69)	.0248	100.0	12.00 (14.31)	<.0001
Complications	No	100.0	2053 (50.59)	1067 (53.40)	.0422	100.0	1331 (37.26)	<.0001
	Yes		2005 (49.41)	931 (46.60)			2241 (62.74)	
Severe complications	No	100.0	3887 (95.79)	1927 (96.45)	.2445	100.0	3342 (93.56)	<.0001
	Yes		171 (4.21)	71 (3.55)			230 (6.44)	
Acute kidney injury	No	100.0	3712 (91.47)	1832 (91.69)	.8121	100.0	2977 (83.34)	<.0001
	Yes		346 (8.53)	166 (8.31)			595 (16.66)	
Laboratory variables								
Arterial base excess	Mean (SD)	97.3	−1.22 (2.32)	−1.26 (2.39)	.0824	99.3	−1.45 (2.95)	<.0001
Arterial hematocrit	Mean (SD)	97.2	29.35 (5.35)	29.33 (5.32)	.5922	99.3	30.78 (5.73)	<.0001
HCO3	Mean (SD)	97.2	23.45 (2.30)	23.43 (2.29)	.4495	99.2	23.74 (2.63)	<.0001
Creatinine	Mean (SD)	97.9	97.70 (51.75)	95.89 (42.59)	.0649	98.8	105.46 (57.06)	<.0001
C-reactive protein	Mean (SD)	80.4	113.22 (77.89)	115.39 (78.48)	.2519	92.7	113.13 (82.37)	.9451
Daily fluid balance	Mean (SD)	81.7	807.13 (860.04)	763.78 (861.34)	.0184	91.9	913.38 (1090.36)	<.0001
Hemoglobin	Mean (SD)	100.0	99.68 (18.01)	99.51 (18.63)	.1740	99.9	100.46 (31.35)	<.0001
Hydrogen ion	Mean (SD)	94.7	40.35 (5.74)	40.37 (6.62)	.7559	98.6	42.93 (17.76)	<.0001
Lactate	Mean (SD)	94.5	1.81 (1.01)	1.82 (1.08)	.3291	97.6	2.01 (1.95)	<.0001
Potassium	Mean (SD)	97.3	4.67 (0.57)	4.67 (0.58)	.5302	99.1	4.64 (0.67)	<.0001
Sodium	Mean (SD)	97.3	136.63 (3.09)	136.67 (3.02)	.1282	99.2	140.35 (390.82)	.0450
Urea	Mean (SD)	81.4	6.53 (2.77)	6.44 (2.68)	.1841	93.9	7.09 (3.66)	<.0001
Urine output	Mean (SD)	88.0	94.47 (80.25)	93.23 (73.08)	.1489	98.4	112.83 (1036.90)	.0053
Medicines								
Vasopressin given	No	100.0	1959 (98.05)	3991 (98.35)	.4621	100.0	3427 (95.94)	<.0001
	Yes		39 (1.95)	67 (1.65)			145 (4.06)	
Noradrenaline given	No	100.0	1228 (61.46)	2473 (60.94)	.7171	100.0	1308 (36.62)	<.0001
	Yes		770 (38.54)	1585 (39.06)			2264 (63.38)	
Dobutamine given	No	100.0	1431 (71.62)	2885 (71.09)	.6919	100.0	2018 (56.49)	<.0001
	Yes		567 (28.38)	1173 (28.91)			1554 (43.51)	
Dopamine given	No	100.0	1899 (95.05)	3831 (94.41)	.3294	100.0	3440 (96.30)	.0001
	Yes		99 (4.95)	227 (5.59)			132 (3.70)	

The *P* values are derived, using χ^2^ tests for categorical and Student *t* tests for numerical variables. *BMI*, Body mass index; *EuroSCORE*, European System for Cardiac Operative Risk Evaluation; *LV*, left ventricular: *NYHA*, New York Heart Association; *MI*, myocardial infarction; *LMS*, left main stem; *PCI*, percutaneous coronary intervention; *CABG*, coronary artery bypass grafting; *ICU*, intensive care unit; *HCO3*, bicarbonate.

**Table E3:** Mean variable importance for the top 10 variables for each lead time for the BARTm model

Variable	Mean importance
Preoperative creatinine	0.0549
Creatinine	0.0296
Urine output	0.0246
Potassium	0.0243
Lactate	0.0238
Urea	0.0228
C-reactive protein	0.0212
Hydrogen ion	0.0203
Hemoglobin	0.0202
Sodium	0.0197
LMS	0.0197
Arterial base excess	0.0193
Vasopressin given	0.0193
Daily fluid balance	0.0188
Sex	0.0181
Age	0.0177
Smoking status	0.0176
Arterial hematocrit	0.0175
Dobutamine given	0.017
Noradrenaline given	0.0167
Logistic EuroSCORE	0.0159
Previous MI	0.0153
Dopamine given	0.0153
Procedure	0.0147
Bicarbonate	0.0147
Extracardiac arteriopathy	0.0137
Previous PCI	0.0132

*LMS*, Left main stem; *EuroSCORE*, European System for Cardiac Operative Risk Evaluation; *MI*, myocardial infarction; *PCI*, percutaneous coronary intervention; *BARTm*, bootstrap aggregated regression trees machine.

**Table E4:** Model coefficients for the logistic regression models for the lead time of 1 to 12 hours before AKI

Variable	1	2	3	4	5	6	7	8	9	10	11	12
(Intercept)	−3.3155	−17.7871	−3.7293	2.6593	8.0087	1.8157	−4.2057	1.4899	−0.3817	6.2444	8.1845	2.1429
ABE min	0.0063	−0.0073	−0.0086	0.0225	0.0061	−0.1343	−0.2274	−0.1271	−0.1769	−0.1204	−0.1088	−0.0234
ABE max	−0.0502	−0.0927	−0.1676	−0.1435	−0.2361	−0.2042	−0.2404	−0.3069	−0.2072	−0.0678	0.0646	−0.0320
ABE first	−0.0904	−0.2155	−0.2909	−0.2211	−0.1323	−0.3108	−0.2389	−0.1128	−0.1417	−0.1822	0.1853	−0.4340
ABE last	0.2710	−0.4662	0.0226	0.2425	0.5614	0.1161	−0.1839	0.4286	0.1529	0.2156	−0.1548	−0.0334
AH min	0.0812	−0.0754	−0.1785	−0.1897	−0.2048	−0.1725	−0.1742	−0.0229	0.0030	−0.0403	−0.0293	−0.1048
AH max	0.0848	−0.1525	−0.1079	−0.1356	−0.1461	−0.1695	−0.1670	−0.2347	−0.2430	−0.2836	−0.1958	−0.0582
AH first	−0.0241	0.1190	0.1111	0.1229	0.1371	0.1507	0.1548	0.1823	0.1854	0.1928	0.1266	0.0380
AH last	−0.0436	0.0918	0.1133	0.1304	0.0447	0.1569	0.1525	0.1276	0.1516	0.1475	−0.0094	0.0940
Creatinine min	−0.1107	−0.1168	−0.1994	−0.2362	−0.2314	−0.3181	−0.2166	9.1287	0.6663	75.3091	159.1650	−2.3706
Creatinine max	−0.2588	−0.1410	−0.3302	−0.3498	−0.3088	−0.2848	−0.2411	9.0462	0.4662	75.1716	158.9959	−12.1159
Creatinine first	0.1209	0.1338	0.2332	0.2669	0.2602	0.3223	0.2439	−9.0750	−0.6089	−75.2371	−159.1060	2.4310
Creatinine last	0.3218	0.1930	0.3585	0.3826	0.3439	0.3367	0.2711	−9.0340	−0.4614	−75.1734	−158.9911	12.1250
CRP min	2.2498	−4.6452	−5.0717	−5.0443	8.4744	−0.0266	−0.0033	11.2194	1.4312	2.3385	2.4364	2.0305
CRP max	−1.5044	−12.7405	−13.8891	−13.8979	−0.0118	−0.0311	−0.0282	−0.0173	−0.0268	−15.4994	−15.6951	−0.7263
CRP first	−2.2390	4.6843	5.0815	5.0522	−8.4616	0.0567	0.0305	−11.2019	−1.4051	13.1606	13.2573	−1.3070
CRP last	1.4936	12.7015	13.8804	13.8913	0.0000	0.0000	0.0000	0.0000	0.0000	0.0000	0.0000	0.0000
DFB min	0.0009	0.0001	0.0014	0.0016	0.0021	0.0011	0.0015	0.0000	−0.0001	0.0001	0.0004	0.0045
DFB max	−0.0006	−0.0009	0.0000	0.0004	0.0009	0.0003	0.0008	−0.0007	−0.0005	−0.0012	−0.0004	0.0021
DFB first	−0.0004	0.0001	−0.0015	−0.0017	−0.0022	−0.0012	−0.0015	−0.0001	0.0000	−0.0004	−0.0003	−0.0046
DFB last	0.0005	0.0010	0.0003	0.0000	−0.0004	0.0001	−0.0004	0.0012	0.0011	0.0018	0.0006	−0.0015
Bicarb min	0.0829	0.1087	0.1240	0.1366	0.1782	0.2363	0.3209	0.2128	0.2834	0.2341	0.1555	0.1526
Bicarb max	0.0492	0.0689	0.0908	0.0959	0.2054	0.1034	0.1306	0.2169	0.2289	0.0488	−0.0269	0.0671
Bicarb first	0.0787	0.1724	0.1845	0.1263	0.0494	0.2472	0.1863	0.0362	0.0368	0.0454	−0.2619	0.2568
Bicarb last	−0.3759	0.2672	−0.0842	−0.3447	−0.6641	−0.2110	0.0670	−0.4465	−0.3182	−0.2749	−0.0199	−0.1322
Hemoglobin min	−0.0337	0.0222	0.0465	0.0477	0.0527	0.0502	0.0533	0.0115	−0.0002	0.0149	0.0221	0.0370
Hemoglobin max	−0.0255	0.0420	0.0377	0.0408	0.0383	0.0459	0.0454	0.0574	0.0745	0.0648	0.0431	0.0270
Hemoglobin first	0.0054	−0.0331	−0.0301	−0.0305	−0.0313	−0.0345	−0.0367	−0.0366	−0.0462	−0.0370	−0.0217	−0.0123
Hemoglobin last	0.0012	−0.0480	−0.0515	−0.0511	−0.0239	−0.0616	−0.0589	−0.0531	−0.0636	−0.0517	−0.0230	−0.0579
Hydrogen ion min	0.0923	0.0385	0.0407	0.0536	0.0534	0.0074	0.0234	0.0489	0.0444	0.0784	0.0764	0.0631
Hydrogen ion max	−0.0260	−0.0153	−0.0032	−0.0020	−0.0031	−0.0161	−0.0228	−0.0345	−0.0351	−0.0158	−0.0328	−0.0168
Hydrogen ion first	−0.0285	−0.0980	−0.1173	−0.0992	−0.0909	−0.1106	−0.1014	−0.0883	−0.0639	−0.0949	0.0341	−0.1181
Hydrogen ion last	0.1399	0.0191	0.0919	0.1126	0.1563	0.0778	−0.0115	0.1638	0.0804	0.0806	−0.0041	0.0411
Lactate min	0.6463	0.2401	0.0637	0.1559	−0.0041	0.0812	0.0654	0.5344	0.3746	−0.0693	0.3309	0.4062
Lactate max	−0.2492	−0.2078	−0.1921	−0.2073	−0.2099	−0.2863	−0.3272	−0.4133	−0.5003	−0.3407	−0.3409	−0.4162
Lactate first	0.2187	0.3597	0.3562	0.3788	0.4005	0.2524	0.2585	0.1586	0.3540	0.4987	0.3028	0.4559
Lactate last	0.4501	0.4529	0.5405	0.4339	0.5316	0.4865	0.4974	0.3769	0.2658	0.2973	0.2935	−0.0251
Potassium min	−0.2827	0.2198	−0.4334	−0.3504	−0.3441	−0.4274	−0.5304	−0.2418	−0.5534	−0.3586	−0.1227	0.1204
Potassium max	0.1336	0.0515	0.0086	0.0009	−0.1053	0.0587	0.0449	−0.0763	−0.3728	−0.3132	−0.3254	−0.3268
Potassium first	−0.4252	−0.0496	0.1751	0.1567	0.2620	0.0009	0.0009	0.0803	0.0691	−0.0094	−0.2962	−0.3284
Potassium last	1.0046	0.3203	0.2132	0.0686	−0.0365	0.2723	0.4531	0.4199	0.6882	0.2802	0.7569	0.6396
Sodium min	−0.0859	−0.0320	−0.0511	−0.0284	−0.0634	−0.0391	−0.0341	−0.0834	−0.0238	−0.0164	−0.0395	0.1001
Sodium max	−0.0599	−0.0681	−0.1123	−0.1048	−0.1013	−0.0957	−0.0995	−0.0633	−0.0651	−0.0557	−0.0626	−0.1983
Sodium first	0.0060	0.0109	0.0403	0.0215	0.0286	0.0157	0.0231	−0.0159	−0.0474	−0.0791	−0.0256	−0.0508
Sodium last	0.1120	0.0775	0.0464	0.0305	0.0507	0.0142	0.0071	0.0682	0.0580	0.0534	0.0315	0.0523
Urea min	11.0466	6.7434	7.2850	5.4943	6.1846	−4.6818	−5.0169	4.0404	−0.2864	165.5889	−393.5287	−10.7716
Urea max	6.6862	4.5250	4.7118	3.2855	3.7942	−6.9378	−5.8298	−117.1044	−8.2502	169.2877	−392.8579	0.4373
Urea first	−9.6053	−5.9957	−6.6836	−4.8759	−5.6490	5.3354	5.4132	−3.7839	0.0301	−165.9517	393.0349	10.5400
Urea last	−7.9795	−5.0604	−5.0433	−3.6405	−4.0421	6.4805	5.6222	117.0219	8.6885	−168.6656	393.5856	0.0000
Urine min	−0.0051	0.0019	0.0030	0.0047	1.0663	1.2276	1.1605	0.0077	0.7161	0.2652	0.1852	0.0081
Urine max	−0.0010	−0.0007	−0.0001	0.0000	−0.1055	0.1532	0.1311	−0.0001	0.2915	0.1883	0.3863	0.0000
Urine first	−0.0010	−0.0005	0.0002	0.0001	0.6010	0.6028	0.6390	−0.0009	0.7481	0.6815	0.5318	−0.0015
Urine last	−0.0012	−0.0005	−0.0006	−0.0001	0.7188	0.6774	0.6153	−0.0021	0.7630	0.6663	0.4355	−0.0032
Vasopressin given	0.6634	0.7241	1.0164	1.0332	0.0115	0.0081	0.0113	0.6302	0.0094	0.0121	0.0075	0.2843
Noradrenaline given	0.5160	0.2441	−0.0958	−0.0976	0.0000	−0.0009	−0.0007	0.1717	−0.0004	−0.0010	−0.0004	0.3262
Dobutamine given	0.5232	0.4145	0.6380	0.6683	0.0001	0.0001	−0.0001	0.7331	−0.0003	0.0005	−0.0003	0.4636
Dopamine given	1.2560	0.7603	0.6814	0.7320	−0.0071	−0.0042	−0.0073	0.6161	−0.0022	−0.0032	−0.0050	0.9150
Sex: female	0.0111	0.3747	0.3938	0.4046	0.4538	0.3205	0.4284	0.3799	0.3606	0.4074	0.5883	0.3347
Logistic EuroSCORE	0.0478	0.0256	0.0232	0.0230	0.0185	0.0081	0.0108	0.0361	0.0393	0.0249	0.0373	0.0228
Priority: emergency	2.1180	2.2483	1.9617	2.0547	2.0984	0.6468	0.7731	1.1973	0.9499	−0.8440	−0.8018	−17.4710
Priority: priority	0.1784	0.2170	0.1567	0.1691	0.1856	0.0346	0.1017	0.0999	−0.1163	0.1337	0.2850	0.0088
Priority: urgent	0.1094	0.1469	0.1602	0.2120	0.0906	0.1895	0.1988	0.2055	0.1048	0.0380	−0.1060	−0.0049
Procedure general: valve	0.3312	0.2453	0.3569	0.3832	0.3595	0.4216	0.3843	0.5319	0.1981	0.3948	0.1701	0.3667
Procedure general: valve and CABG	0.1346	0.0128	−0.0375	−0.0301	0.0026	0.1510	0.1554	0.2010	−0.0663	0.1208	0.0674	0.3790
LV function: moderate	0.1271	0.1472	−0.0098	0.0148	0.0047	−0.2093	−0.2480	0.1120	−0.2013	−0.1196	−0.1577	−0.1431
LV function: poor	1.0494	0.9072	0.6258	0.7121	0.6841	0.4534	0.4881	0.6449	−0.1105	0.0090	0.3895	1.2613
NYHA grade: II	0.0098	0.1272	0.0232	−0.0049	−0.0523	0.0117	0.0392	0.2271	−0.1424	−0.0176	0.0627	−0.1558
NYHA grade: III	0.3539	0.5042	0.1829	0.1589	0.1206	0.2039	0.2755	0.1627	−0.1599	0.0097	0.0432	−0.2152
NYHA grade: IV	0.9812	0.5170	0.1903	0.2548	0.3044	0.3213	0.3727	0.2427	0.1805	0.3143	0.1702	0.3641
Angina status: I	−0.3182	−0.1685	−0.3666	−0.4249	−0.4512	−0.3464	−0.3428	−0.0656	−0.2999	−0.5215	−0.5303	−0.2028
Angina status: II	−0.3681	−0.5307	−0.5056	−0.4663	−0.4706	−0.4504	−0.4352	−0.0442	−0.2702	−0.3509	−0.4239	−0.3714
Angina status: III	−0.3278	−0.4556	−0.3121	−0.3384	−0.3908	−0.2554	−0.2472	−0.0198	−0.1007	−0.1691	−0.2199	−0.7443
Angina status: IV	−0.1793	−0.7894	−0.5899	−0.6593	−0.6113	−0.4667	−0.4195	−0.2024	−0.7655	−0.6598	−0.3888	−1.3840
Renal impairment: moderate	0.3586	0.3428	0.1822	0.1761	0.1599	0.1146	0.0702	0.2520	0.2362	0.3592	0.3446	0.4735
Renal impairment: severe	0.6327	0.3185	0.3561	0.3658	0.2970	0.5425	0.4306	0.4375	0.4401	0.7055	0.7157	1.1448
Renal impairment: unknown	−0.0267	−0.1335	0.1924	0.2272	0.2427	0.3367	0.2797	0.2650	0.0405	0.5162	0.9252	0.0090
Rhythm: abnormal	0.1730	0.0090	0.1172	0.0700	0.0724	0.0074	0.0298	−0.0252	0.1157	0.1015	0.0563	0.1501
Rhythm: unknown	−0.5016	−0.3388	−0.3636	−0.3592	−0.2010	−0.0928	−0.0248	0.1615	0.2421	−0.3630	−0.0779	−0.2895
Previous operations	−0.2423	−0.0787	−0.1689	−0.2262	−0.2951	0.0581	0.0886	−0.6988	−0.7157	−0.2419	0.4732	−0.8792
Neurologic dysfunction	−17.7479	−14.4475	−14.4227	−14.5516	−17.5575	−13.6783	−13.8796	−20.2477	−16.0784	−23.5500	−22.7321	−19.8565
Smoking status: ex-smoker	−0.0831	0.1362	−0.0741	−0.0483	−0.0097	−0.1456	−0.1622	−0.2579	−0.1638	−0.2439	−0.0752	−0.2594
Smoking status: current smoker	−0.6165	−0.3329	−0.6959	−0.7270	−0.7993	−0.6106	−0.6613	−0.2541	−0.7612	−0.3738	−0.6334	−0.1216
Smoking status: unknown	0.1196	0.0189	−0.0970	−0.0470	−0.0222	−0.2234	−0.2392	−0.1354	−0.1247	−0.1752	0.0740	−0.3442
Previous MI	0.3932	0.1360	0.3796	0.3530	0.3549	0.2792	0.2435	0.2233	0.1359	0.3021	0.4534	0.4075
LMS	0.0215	−0.3172	−0.2956	−0.2942	−0.3061	−0.0987	−0.0954	−0.4625	−0.5473	−0.4215	−0.2026	0.1881
LMS: unknown	−0.5476	−0.3519	−0.4491	−0.4678	−0.4728	−0.2828	−0.2426	−0.2407	−0.5953	−0.3579	−0.4574	−0.3244
Preoperative creatinine	−0.0831	−0.0796	−0.0714	−0.0741	−0.0750	−0.0671	−0.0653	−0.0715	−0.0666	−0.0750	−0.0721	−0.0778
Pulmonary disease	−0.0917	0.1811	0.2191	0.2539	0.2366	0.2267	0.1431	0.1687	0.2698	0.2112	0.3381	0.3158
Hypertension history	0.1187	0.1256	0.0837	0.1005	0.1139	−0.0226	0.0033	0.0972	0.0304	0.1703	0.2109	0.0118
Congestive cardiac failure	−1.0520	−0.7182	−0.5117	−0.4874	−0.4518	−0.3109	−0.3438	−0.5262	−0.5975	−0.9438	−0.8085	−0.8693
Previous PCI	0.3554	−0.0045	0.1172	0.1411	0.1590	0.3459	0.3808	0.4925	0.3935	0.3369	0.3103	0.1592
Extracardiac arteriopathy	−0.0399	0.4178	0.3839	0.3372	0.3786	0.5793	0.5605	0.4742	0.1821	0.2177	−0.0029	0.0057
Critical preoperative state	−2.2082	−0.7259	−0.8993	−1.0321	−0.8515	−0.5435	−0.4020	−1.0670	−0.5831	−1.0219	−0.3391	−0.5206
Diabetes	0.1522	0.2734	0.2711	0.2763	0.2816	0.3002	0.2822	0.3153	0.3739	0.2150	0.5108	0.3653
BMI: 25.1-30.0	−0.1371	0.3601	0.3667	0.3783	0.3637	0.4162	0.3739	0.1674	0.0524	0.3175	0.2275	0.3903
BMI: >30.0	−0.1909	−0.0155	0.2896	0.2808	0.2874	0.2632	0.2250	0.0815	−0.0258	0.0461	0.3039	0.1158
Age: 61-67 y	0.0982	0.6297	0.6889	0.6465	0.5612	0.7044	0.7588	0.6369	0.3446	0.2626	0.4007	0.3470
Age: 68-74 y	−0.1550	0.5128	0.7029	0.6289	0.5352	0.6867	0.7007	0.5147	0.1769	0.2735	0.4187	0.0735
Age: 75-99 y	−0.1484	0.5417	0.6389	0.6115	0.5487	0.8551	0.8870	0.6303	0.4801	0.3903	0.3544	0.3606
Active endocarditis	1.8186	1.5886	0.9655	0.9881	1.1905	1.9725	1.7755	1.1651	−0.2248	1.1158	0.9636	1.0412

*ABE*, Arterial base excess; *AH*, arterial hematocrit; *CRP*, C-reactive protein; *DFB*, daily fluid balance; *EuroSCORE*, European System for Cardiac Operative Risk Evaluation; *CABG*, coronary artery bypass grafting; *LV*, left ventricular; *NYHA*, New York Heart Association; *MI*, myocardial infarction; *LMS*, left main stem; *PCI*, percutaneous coronary intervention; *BMI*, body mass index; *AKI*, acute kidney injury.

**Table E5:** Model coefficients for the logistic regression models for the lead time of 13 to 24 hours before AKI

Variable	13	14	15	16	17	18	19	20	21	22	23	24
(Intercept)	11.1764	−1.4593	−1.8628	−3.7877	−1.9712	4.0283	−4.0620	1,958,193,855,762,030.0	5,585,119,126,415,140.0	4.8024	1.7825	0.7851
ABE min	−0.2027	−0.0609	−0.1766	−0.2534	−0.1558	−0.1387	−0.2326	13,858,619,475,796.9	350,900,481,015,319.0	0.3286	0.4133	0.0988
ABE max	−0.2021	−0.0423	−0.2011	−0.1413	−0.1125	0.0259	0.0129	111,177,907,885,775.0	−29,255,273,696,403.4	0.4522	0.1248	−0.4461
ABE first	0.0470	−0.1084	0.0438	0.1277	−0.0081	0.0033	0.1000	−38,277,316,290,424.4	−98,856,639,379,121.9	−0.6334	−0.1779	−0.9056
ABE last	0.4039	−0.2183	0.0655	−0.2006	−0.0429	−0.1153	−0.2067	−143,035,041,460,717.0	52,244,765,191,140.9	−0.4281	−0.7057	1.1877
AH min	−0.0671	−0.0749	−0.1040	−0.0668	0.0065	0.0943	−0.0376	559,486,030,361.7	−57,006,622,066,613.7	−0.0684	−0.1692	0.3181
AH max	−0.1065	−0.0943	−0.2045	−0.0381	−0.1015	−0.1125	−0.1408	−113,914,217,742,307.0	−26,461,413,585,431.2	−0.0793	0.0225	0.8945
AH first	0.0718	0.0864	0.1453	0.0467	0.0651	0.0740	0.1192	111,886,078,822,010.0	9,931,408,211,317.1	0.0516	0.0489	−0.7051
AH last	0.0309	0.0597	0.0617	0.0318	0.0580	−0.0259	0.0316	43,201,926,222,481.1	95,186,622,451,119.5	0.1387	0.1193	−0.5107
Creatinine min	−35.4011	−3.9604	−20.8515	−8.9203	10.7133	0.0137	−0.0652	−60,420,211,285,212.2	341,086,840,399,494.0	3.2495	−1.2560	1.4906
Creatinine max	−35.4307	−7.3655	−20.8573	−19.5405	0.2084	0.1408	0.2404	−35,124,103,106,448.5	242,637,986,072,914.0	0.9891	1.3218	−3.5984
Creatinine first	35.4384	3.9680	20.9150	9.0896	−10.8580	−0.0928	−0.1003	110,035,513,622,027.0	−574,941,590,667,754.0	−4.1716	0.0000	2.1689
Creatinine last	35.4595	7.4180	20.8601	19.4381	0.0000	0.0000	0.0000	0.0	0.0	0.0000	0.0000	0.0000
CRP min	2.3216	1.8825	2.4004	1.5322	2.3712	3.5366	2.1882	733,642,171,992,321.0	−161,986,286,593,525.0	−1.7777	−2.1667	−0.2960
CRP max	−13.3124	−2.7804	−7.7058	−3.3780	−6.9967	−9.8809	−11.3768	−19,515,989,893,487.2	25,949,766,394,516.4	0.0297	0.0546	0.2960
CRP first	10.9907	0.8959	5.3064	1.8426	4.6256	6.3480	9.1923	−709,403,652,543,203.0	136,197,162,492,844.0	1.7491	2.1147	0.0000
CRP last	0.0000	0.0000	0.0000	0.0000	0.0000	0.0000	0.0000	0.0	0.0	0.0000	0.0000	0.0000
DFB min	0.0037	0.0023	0.0019	−0.0488	1.9347	−0.8345	1.3245	3,702,651,213,109.8	1,506,564,672,025.5	0.0225	0.0117	1.1226
DFB max	0.0029	0.0013	0.0011	−0.1645	1.8549	−2.1152	−0.0013	497,494,511,277.5	−646,707,032,013.5	0.0005	0.0003	−0.0932
DFB first	−0.0038	−0.0020	−0.0020	0.0488	−1.9352	0.8381	−1.3230	−1,497,788,972,514.8	−725,422,913,808.8	−0.0226	−0.0117	−1.0291
DFB last	−0.0025	−0.0010	−0.0006	0.1649	−1.8541	2.1119	0.0000	−2,369,861,095,353.3	0.0	0.0000	0.0000	0.0000
Bicarb min	0.2842	0.1636	0.3158	0.3189	0.2417	0.1698	0.1671	−23,774,107,698,308.2	−16,906,470,964,048.6	−0.2274	−0.2705	0.0199
Bicarb max	0.0705	−0.1022	0.0183	0.0243	−0.0525	−0.0103	−0.0228	−108,908,906,183,672.0	−55,504,864,731,627.0	−0.5262	−0.3094	−0.2326
Bicarb first	0.0232	0.1234	−0.0063	−0.0810	0.0675	−0.0325	−0.0793	67,877,784,772,170.6	100,217,193,715,025.0	0.5324	0.1897	0.9803
Bicarb last	−0.4839	0.0431	−0.1692	0.0834	−0.0482	−0.0149	0.0822	79,807,883,722,227.5	−245,965,630,382,425.0	0.3920	0.6020	−0.7513
Hemoglobin min	0.0281	0.0380	0.0335	0.0300	0.0016	−0.0130	0.0092	−8,158,168,901,732.0	−17,228,905,555,540.0	0.0004	0.0475	−0.0330
Hemoglobin max	0.0136	0.0209	0.0376	0.0086	0.0313	0.0154	0.0208	34,842,063,050,603.2	−37,244,568,853,888.4	0.0219	0.0028	−0.1511
Hemoglobin first	−0.0058	−0.0248	−0.0280	−0.0151	−0.0248	−0.0131	−0.0183	−36,535,384,140,446.5	36,879,969,370,636.9	−0.0087	−0.0313	0.0981
Hemoglobin last	−0.0250	−0.0303	−0.0227	−0.0208	−0.0259	−0.0206	−0.0156	−9,856,205,554,322.7	18,096,519,964,225.9	−0.0342	−0.0279	0.0726
Hydrogen Ion min	0.0022	0.0474	0.0607	0.0665	0.0506	−0.0633	−0.0234	25,014,613,370,127.8	34,983,554,507,504.0	0.1387	0.1073	0.0338
Hydrogen ion max	−0.0660	−0.0244	−0.0214	−0.0452	−0.0375	−0.0547	−0.1228	−15,489,630,419,108.8	−17,943,875,401,800.7	−0.0665	0.0274	−0.0428
Hydrogen ion first	0.0052	−0.0380	−0.0347	−0.0296	−0.0621	0.0160	0.0344	−17,331,077,301,091.5	−24,989,092,546,939.1	−0.1387	−0.0760	−0.2579
Hydrogen ion last	0.1353	−0.0299	0.0101	−0.0010	0.0322	0.0506	0.0357	1,881,982,348,406.3	58,758,525,345,303.9	0.0189	−0.1978	0.2476
Lactate min	0.4977	0.1530	−0.1672	0.0039	−0.4495	−0.4509	−0.2116	119,530,414,807,721.0	20,342,538,808,739.6	0.2384	−3.0192	−0.7430
Lactate max	−0.4111	−0.4492	−0.2537	−0.2567	−0.2838	−0.3994	−0.2024	−395,314,486,873,560.0	−437,870,350,166,224.0	−0.9958	−5.3638	−6.7972
Lactate first	0.3242	0.5715	0.6264	0.4421	0.6207	0.7175	0.4969	393,116,263,917,665.0	243,249,464,535,678.0	0.8582	4.2553	5.5431
Lactate last	−0.0269	0.0903	0.0385	−0.1425	0.0649	0.1122	−0.1192	30,206,347,589,401.1	265,316,107,716,496.0	0.1037	4.3038	2.0882
Potassium min	−0.1330	−0.0941	−0.2098	−0.2086	−0.2673	−0.0546	−0.7120	−520,965,815,989,114.0	−127,711,665,848,273.0	−0.1967	−1.5767	0.8270
Potassium max	−0.2287	−0.2979	−0.4505	−0.2292	−0.0919	−0.2713	−0.1820	−332,092,600,672,306.0	−220,862,612,157,605.0	−0.5525	−1.5560	0.5471
Potassium first	−0.1165	−0.1173	0.0420	0.0576	0.1689	0.2707	0.4109	187,025,784,415,029.0	45,660,344,765,347.2	0.0857	1.4827	−0.1776
Potassium last	0.0659	0.3917	0.1502	0.2037	0.1523	0.0318	0.4171	435,387,696,858,357.0	216,762,379,616,276.0	0.3854	1.3794	−1.3217
Sodium min	0.0791	0.0532	0.1054	0.0526	0.1101	0.0734	0.2118	88,115,150,907,139.7	−17,800,701,837,982.3	0.1435	0.1202	−0.0932
Sodium max	−0.0061	−0.0174	0.0001	0.0131	−0.1297	−0.1865	−0.1168	−35,942,161,734,412.9	−20,719,522,776,091.3	−0.0269	0.0494	0.0156
Sodium first	−0.0712	−0.0027	−0.0426	−0.0343	0.0032	0.1098	−0.0437	−31,177,778,205,981.0	−35,361,720,840,845.8	−0.1333	−0.1524	0.0621
Sodium last	−0.1081	−0.0832	−0.0982	−0.0858	−0.0270	−0.0531	−0.0608	−44,009,293,290,976.0	35,519,006,039,801.4	−0.0553	−0.0611	−0.0222
Urea min	0.4752	52.0079	155.4182	−15.5058	0.6726	154.2554	147.6371	−296,585,365,536,571.0	18,287,964,416,913,400.0	83.0758	91.6785	0.1886
Urea max	0.8804	0.2852	0.7525	2.3355	−1.4322	1.5103	5.9762	2,701,948,735,017,500.0	−10,223,020,053,755,000.0	−52.5660	−61.1843	0.0000
Urea first	−1.1951	−52.1130	−156.0342	13.3238	0.9397	−155.5679	−153.3898	−2,354,912,493,860,920.0	−7,962,293,744,809,060.0	−30.3409	−30.2719	0.0000
Urea last	0.0000	0.0000	0.0000	0.0000	0.0000	0.0000	0.0000	0.0	0.0	0.0000	0.0000	0.0000
Urine min	0.9824	0.9920	0.3231	−0.0012	0.9442	0.3870	−0.0726	4,166,049,346,988.0	67,491,967,728,257.8	−0.1699	0.4835	−0.0069
Urine max	0.3417	0.2453	0.5359	−0.0004	0.1510	0.1605	0.3300	−6,256,305,204.0	6,372,718,826,092.3	0.1020	0.3244	−0.0138
Urine first	0.6157	0.5004	0.5354	0.0000	0.5857	0.5733	0.4173	−582,802,469,257.0	174,665,332,726,455.0	0.4910	0.4258	0.0121
Urine last	0.8789	0.6744	0.7473	0.0010	0.7615	0.7600	0.4828	−1,613,716,817,827.4	161,251,664,239,996.0	1.0589	0.8050	0.0086
Vasopressin given	0.0070	0.0004	0.0031	0.9550	0.0019	0.0082	0.0064	−660,476,569,970,552.0	−927,670,247,671.5	−0.0006	−0.0008	0.1193
Noradrenaline given	−0.0006	0.0000	0.0000	0.3777	−0.0003	0.0000	−0.0002	120,601,601,771,668.0	−335,737,741,226.9	−0.0021	−0.0056	0.3045
Dobutamine given	−0.0002	−0.0004	−0.0015	0.6238	−0.0005	−0.0007	−0.0003	323,602,996,948,020.0	−279,264,256,744.0	0.0008	0.0035	0.4275
Dopamine given	0.0003	0.0008	−0.0011	0.7192	0.0023	−0.0045	−0.0008	303,941,959,026,546.0	1,159,593,580,102.6	0.0022	0.0022	0.7383
Sex: female	0.4622	0.3150	0.2985	0.6241	0.6448	0.3086	0.5998	345,371,277,098,159.0	76,495,231,702,591.2	0.3047	0.3693	0.2149
Logistic EuroSCORE	0.0107	0.0117	0.0175	0.0049	0.0204	−0.0235	−0.0318	−30,343,418,378,487.5	30,710,037,795,255.1	−0.0080	0.0033	−0.0221
Priority: emergency	0.6943	1.3087	0.8511	−17.6079	−18.6589	−23.8863	−23.4192	−1,971,847,600,283,780.0	322,747,931,080,681.0	−14.3587	−13.6796	−18.1191
Priority: priority	0.0235	0.2849	−0.4391	0.0034	−0.0568	0.2481	0.2195	316,053,159,768,962.0	25,974,563,805,617.0	0.1343	0.2463	−0.0891
Priority: urgent	0.2012	−0.0430	−0.0941	−0.0798	−0.4309	0.1641	0.0204	4,326,974,520,148.6	223,352,253,391,998.0	−0.2024	0.0485	−0.2259
Procedure general: valve	0.6321	0.6466	0.3951	0.3021	0.3868	0.6366	0.7870	341,182,694,636,460.0	−3,218,030,512,994.7	0.6832	0.7335	0.7634
Procedure general: valve and CABG	0.6461	0.6296	0.5693	0.4611	0.4727	0.3689	0.7741	352,467,708,701,108.0	−155,845,346,944,126.0	0.5084	0.6441	0.6282
LV function: moderate	0.0787	0.1188	−0.0747	−0.0417	−0.0142	0.0923	0.4501	82,124,446,920,578.9	17,751,823,594,108.0	0.0045	−0.2407	0.1936
LV function: poor	0.4991	1.1058	0.6683	0.6856	0.6722	1.3484	1.6069	787,410,410,213,189.0	−132,432,303,771,795.0	0.9261	0.7524	1.1662
NYHA grade: II	−0.1590	−0.1184	−0.2403	−0.1286	−0.1617	−0.2357	−0.5153	−273,820,184,106,736.0	−16,009,053,969,651.9	−0.3722	−0.1615	−0.0168
NYHA grade: III	−0.2379	0.0042	−0.1555	−0.1488	−0.0495	0.0182	−0.3463	−258,273,913,499,150.0	120,322,027,532,904.0	−0.3584	−0.0142	−0.0654
NYHA grade: IV	0.1427	0.0813	0.0098	0.0724	0.4137	0.3725	−0.5374	−23,589,275,230,276.4	−285,245,892,451,566.0	−0.4942	0.1386	0.4162
Angina status: I	−0.3308	−0.0480	−0.3020	−0.6292	−0.2673	0.0654	−0.0308	−69,394,199,112,350.3	−288,450,109,165,304.0	−0.0851	0.0627	0.1309
Angina status: II	−0.2382	0.0592	0.0518	−0.1923	0.0579	−0.0310	0.1332	−57,796,223,173,130.6	−21,272,276,019,237.1	0.0559	0.1731	0.3038
Angina status: III	−0.2105	−0.3501	−0.2411	−0.3826	−0.0736	−0.0650	0.1882	−5,113,282,631,469.2	−200,277,983,846,487.0	0.1550	0.3368	0.2153
Angina status: IV	−0.7431	−0.9675	−0.4427	−1.1802	−1.1594	−1.8303	−0.3981	−824,762,954,162,916.0	−177,683,211,365,535.0	−0.0286	−0.6198	−0.2265
Renal impairment: moderate	0.3499	0.1594	0.1969	0.3361	0.2659	0.3260	0.1471	3,996,953,666,564.7	11,287,267,767,735.9	0.5724	0.3685	−0.1267
Renal impairment: severe	0.8230	0.8780	0.7199	0.5221	0.6694	0.8190	0.5860	401,551,636,039,549.0	−453,087,903,256,738.0	1.2477	0.8375	0.2642
Renal impairment: unknown	0.4947	0.1430	0.4336	0.1413	−0.1538	0.0557	0.3535	17,502,129,088,324.6	12,798,642,332,972.7	0.6687	0.6509	−0.1074
Rhythm: abnormal	0.0495	−0.0729	0.1356	0.4073	0.3323	0.2433	0.2428	279,937,961,386,447.0	63,209,234,110,804.0	0.2783	0.1217	0.1148
Rhythm: unknown	−0.3791	−0.5700	0.0522	0.5768	−0.2171	−0.5300	0.1680	60,798,488,872,892.8	−26,927,516,718,715.6	−0.5568	−0.5909	−0.0547
Previous operations	0.0692	−0.0621	−0.6363	−0.4417	−0.6379	−0.2952	−0.3003	−708,595,443,801,867.0	−756,412,101,465,413.0	−0.8198	−1.8197	0.1198
Neurologic dysfunction	−20.4808	−19.4998	−25.2036	−22.4815	−18.9833	−24.4765	−23.9815	−1,008,136,057,294,050.0	−182,028,864,286,183.0	−15.1816	−15.0233	−18.7356
Smoking status: ex-smoker	−0.2614	−0.1024	0.0765	−0.1202	−0.0776	−0.3629	−0.4601	−69,931,265,151,681.5	142,607,682,712,594.0	0.1451	0.1877	−0.0767
Smoking status: current smoker	−0.3683	−0.2210	−0.4705	−0.3746	−0.6081	−0.8252	−0.4506	−575,741,341,061,196.0	34,465,434,416,503.7	−0.2477	−0.4088	−0.4299
Smoking status: unknown	−0.1427	0.2083	0.0391	−0.0885	0.0670	−0.1887	−0.2752	−155,333,467,019,895.0	−99,389,855,535,395.4	0.2220	0.0166	−0.3057
Previous MI	0.3978	0.2582	0.2702	0.4305	0.5803	0.5596	0.6301	494,907,310,513,025.0	34,812,436,243,184.6	0.5215	0.6433	0.3551
LMS	−0.0870	0.2028	−0.0129	−0.2164	−0.1284	0.0346	0.0261	35,065,534,984,794.8	−196,494,725,372,545.0	−0.5632	−0.3964	−0.4696
LMS: unknown	−0.1831	−0.3757	−0.1683	−0.7333	−0.4563	−0.3966	−0.4505	−310,770,720,379,790.0	−174,580,331,014,328.0	−0.5381	−0.5337	−0.4635
Preoperative creatinine	−0.0752	−0.0686	−0.0728	−0.0712	−0.0703	−0.0669	−0.0809	−16,137,640,218,124.3	−2,612,550,702,842.2	−0.0740	−0.0735	−0.0650
Pulmonary disease	0.4828	0.2156	0.1504	0.3204	0.3570	0.4784	0.6384	414,512,907,849,904.0	−98,530,520,254,170.0	0.6291	0.3078	0.3319
Hypertension history	−0.1455	−0.0133	−0.0663	0.0443	−0.2285	0.1058	−0.0274	332,077,287,266,487.0	199,961,290,995,373.0	−0.0482	−0.0791	0.2475
Congestive cardiac failure	−0.9605	−0.6768	−0.5933	−0.3865	−0.7661	−0.7136	−0.5393	−104,873,226,391,883.0	−450,357,512,685,601.0	−0.3778	−0.5893	−0.4518
Previous PCI	0.3168	0.3849	0.2414	0.1079	0.0233	0.3640	0.2901	226,201,046,071,099.0	48,717,479,884,320.2	0.0337	0.1500	0.3677
Extracardiac arteriopathy	0.8146	0.4963	0.5438	0.2736	0.3462	0.7859	0.6302	378,547,596,618,020.0	−88,230,216,064,613.6	0.5492	0.5695	0.6683
Critical preoperative state	−0.3347	−0.0175	−0.6026	−0.3866	−0.4886	−0.5433	−0.4305	22,650,172,629,481.7	−558,323,660,364,674.0	−0.0414	−1.0724	0.8801
Diabetes	0.4135	0.2664	0.1325	0.0914	0.1200	0.0935	0.1579	122,126,489,813,521.0	−59,795,625,718,223.5	0.2252	0.0347	−0.0624
BMI: 25.1-30.0	0.0435	0.1589	0.5661	0.0652	0.2376	0.3211	0.2715	100,321,949,563,768.0	−132,231,973,653,146.0	0.1474	0.2482	0.1715
BMI: >30.0	−0.1002	0.1692	0.1882	0.1109	−0.0796	0.2415	0.1388	−57,675,093,738,626.0	−45,193,595,921,922.3	−0.0626	0.1882	0.3477
Age: 61-67 y	0.2920	0.4467	0.2995	0.5269	0.3019	0.2168	0.4195	235,509,488,773,289.0	196,116,064,124,413.0	0.3749	0.7335	0.7166
Age: 68-74 y	0.1486	0.2835	0.0562	0.3268	0.2514	0.0030	0.4656	347,695,477,943,977.0	−126,564,424,967,840.0	0.2194	0.6978	0.6539
Age: 75-99 y	0.2943	0.4738	0.4273	0.5840	0.4108	0.1258	0.5106	419,108,620,156,596.0	140,362,598,360,892.0	0.5202	0.9097	1.3133
Active endocarditis	1.0306	1.0274	0.7359	1.2976	0.9216	−0.4330	−0.1379	−165,656,675,206,947.0	−2,188,665,690,240,340.0	1.1748	1.3240	0.9812

*ABE*, Arterial base excess; *AH*, arterial hematocrit; *CRP*, C-reactive protein; *DFB*, daily fluid balance; *EuroSCORE*, European System for Cardiac Operative Risk Evaluation; *CABG*, coronary artery bypass grafting; *LV*, left ventricular; *NYHA*, New York Heart Association; *MI*, myocardial infarction; *LMS*, left main stem; *PCI*, percutaneous coronary intervention; *BMI*, body mass index; *AKI*, acute kidney injury; .

**Table E6:** Performance measures for each model for each lead time applied on each dataset when predicting the onset of AKI within 25 hours, up to 24 hours in advance in ICU

Lead time	AUC	Sensitivity	Specificity	PPV	NPV	Cut-Off
Training data						
BARTm						
−1	0.954	0.918	0.864	0.010	0.593	0.128
−2	0.935	0.922	0.782	0.009	0.710	0.092
−3	0.916	0.918	0.747	0.010	0.741	0.078
−4	0.929	0.849	0.850	0.017	0.647	0.126
−5	0.919	0.927	0.748	0.009	0.738	0.079
−6	0.927	0.919	0.780	0.010	0.711	0.097
−7	0.929	0.923	0.766	0.010	0.722	0.091
−8	0.919	0.898	0.773	0.012	0.726	0.094
−9	0.926	0.875	0.825	0.015	0.669	0.117
−10	0.914	0.817	0.841	0.021	0.670	0.130
−11	0.918	0.792	0.880	0.021	0.624	0.152
−12	0.929	0.876	0.846	0.130	0.664	0.121
−13	0.923	0.912	0.791	0.009	0.729	0.094
−14	0.901	0.892	0.767	0.012	0.753	0.088
−15	0.928	0.859	0.858	0.013	0.669	0.126
−16	0.923	0.883	0.825	0.012	0.705	0.109
−17	0.903	0.815	0.830	0.017	0.726	0.113
−18	0.915	0.783	0.882	0.019	0.661	0.134
−19	0.924	0.871	0.822	0.012	0.731	0.098
−20	0.925	0.863	0.842	0.012	0.703	0.110
−21	0.926	0.880	0.818	0.011	0.737	0.094
−22	0.925	0.871	0.829	0.012	0.721	0.102
−23	0.926	0.838	0.871	0.014	0.675	0.123
−24	0.909	0.895	0.783	0.090	0.782	0.077
Mean (SD)						
Logistic regression						
−1	0.944	0.957	0.789	0.006	0.684	0.056
−2	0.915	0.872	0.799	0.015	0.705	0.073
−3	0.905	0.826	0.831	0.020	0.680	0.094
−4	0.903	0.817	0.836	0.021	0.675	0.102
−5	0.904	0.826	0.828	0.020	0.684	0.095
−6	0.889	0.765	0.852	0.026	0.666	0.113
−7	0.892	0.787	0.843	0.024	0.672	0.110
−8	0.905	0.792	0.862	0.022	0.647	0.119
−9	0.897	0.835	0.800	0.020	0.708	0.086
−10	0.900	0.844	0.803	0.018	0.707	0.081
−11	0.901	0.797	0.849	0.021	0.675	0.109
−12	0.911	0.836	0.836	0.017	0.689	0.088
−13	0.890	0.825	0.803	0.018	0.736	0.075
−14	0.888	0.784	0.836	0.022	0.709	0.094
−15	0.888	0.751	0.874	0.023	0.672	0.119
−16	0.892	0.793	0.846	0.020	0.700	0.102
−17	0.883	0.815	0.793	0.018	0.763	0.073
−18	0.881	0.743	0.865	0.022	0.701	0.112
−19	0.898	0.747	0.886	0.021	0.671	0.119
−20	0.648	0.326	0.970	0.051	0.544	0.000
−21	0.515	0.036	0.994	0.067	0.684	0.000
−22	0.888	0.813	0.819	0.017	0.746	0.077
−23	0.903	0.862	0.796	0.013	0.762	0.065
−24	0.894	0.791	0.848	0.016	0.740	0.086
Mean (SD)						
Testing data						
BARTm						
−1	0.918	0.932	0.764	0.008	0.734	0.082
−2	0.876	0.791	0.833	0.025	0.671	0.115
−3	0.881	0.816	0.814	0.023	0.690	0.104
−4	0.887	0.868	0.765	0.017	0.726	0.088
−5	0.887	0.798	0.837	0.024	0.665	0.121
−6	0.871	0.865	0.726	0.018	0.761	0.081
−7	0.876	0.811	0.789	0.023	0.723	0.101
−8	0.851	0.812	0.756	0.024	0.749	0.084
−9	0.838	0.730	0.801	0.029	0.754	0.110
−10	0.848	0.767	0.788	0.027	0.750	0.105
−11	0.837	0.837	0.723	0.021	0.780	0.069
−12	0.823	0.832	0.686	0.022	0.806	0.053
−13	0.832	0.743	0.769	0.030	0.774	0.092
−14	0.830	0.840	0.700	0.019	0.809	0.063
−15	0.814	0.723	0.753	0.030	0.802	0.084
−16	0.837	0.855	0.693	0.015	0.829	0.061
−17	0.856	0.789	0.749	0.022	0.797	0.077
−18	0.843	0.795	0.739	0.021	0.806	0.069
−19	0.837	0.813	0.729	0.021	0.803	0.066
−20	0.831	0.857	0.693	0.015	0.826	0.059
−21	0.817	0.793	0.719	0.022	0.819	0.054
−22	0.841	0.840	0.698	0.016	0.832	0.063
−23	0.843	0.886	0.651	0.012	0.847	0.043
−24	0.832	0.914	0.601	0.010	0.857	0.036
Mean (SD)						
Logistic regression						
−1	0.896	0.825	0.841	0.019	0.678	0.078
−2	0.847	0.757	0.822	0.030	0.695	0.085
−3	0.862	0.860	0.789	0.018	0.706	0.075
−4	0.858	0.798	0.793	0.025	0.717	0.080
−5	0.855	0.781	0.814	0.027	0.699	0.089
−6	0.887	0.766	0.854	0.027	0.657	0.120
−7	0.867	0.793	0.813	0.025	0.704	0.093
−8	0.841	0.679	0.863	0.036	0.667	0.125
−9	0.857	0.720	0.838	0.029	0.717	0.107
−10	0.856	0.786	0.792	0.024	0.741	0.077
−11	0.840	0.817	0.708	0.024	0.793	0.051
−12	0.761	0.624	0.765	0.043	0.806	0.055
−13	0.827	0.663	0.872	0.034	0.679	0.125
−14	0.810	0.670	0.821	0.033	0.760	0.094
−15	0.800	0.766	0.710	0.027	0.818	0.050
−16	0.802	0.807	0.654	0.021	0.852	0.037
−17	0.824	0.556	0.940	0.037	0.573	0.190
−18	0.796	0.648	0.801	0.034	0.795	0.076
−19	0.585	0.209	0.961	0.063	0.694	0.000
−20	0.529	0.083	0.975	0.200	0.934	0.000
−21	0.552	0.138	0.966	0.065	0.760	0.000
−22	0.839	0.728	0.823	0.023	0.770	0.080
−23	0.830	0.759	0.789	0.021	0.797	0.064
−24	0.838	0.802	0.769	0.018	0.798	0.057
Mean (SD)						
Validation data						
BARTm						
−1	0.907	0.824	0.873	0.016	0.655	0.127
−2	0.886	0.774	0.844	0.021	0.712	0.114
−3	0.855	0.755	0.847	0.023	0.715	0.135
−4	0.860	0.814	0.783	0.019	0.767	0.113
−5	0.844	0.814	0.744	0.020	0.795	0.090
−6	0.864	0.682	0.902	0.028	0.640	0.215
−7	0.851	0.767	0.821	0.022	0.743	0.143
−8	0.828	0.788	0.753	0.022	0.797	0.108
−9	0.814	0.765	0.755	0.024	0.800	0.106
−10	0.821	0.791	0.787	0.021	0.773	0.108
−11	0.811	0.818	0.684	0.020	0.834	0.072
−12	0.835	0.793	0.814	0.019	0.753	0.102
−13	0.837	0.758	0.842	0.021	0.731	0.151
−14	0.834	0.780	0.780	0.020	0.792	0.100
−15	0.841	0.759	0.851	0.020	0.727	0.147
−16	0.824	0.817	0.760	0.017	0.801	0.101
−17	0.824	0.764	0.849	0.018	0.746	0.109
−18	0.864	0.746	0.892	0.018	0.694	0.162
−19	0.858	0.785	0.862	0.016	0.734	0.140
−20	0.842	0.881	0.711	0.010	0.839	0.061
−21	0.825	0.826	0.780	0.014	0.810	0.089
−22	0.824	0.901	0.697	0.009	0.846	0.052
−23	0.879	0.747	0.888	0.017	0.714	0.158
−24	0.834	0.799	0.834	0.014	0.778	0.110
Mean (SD)						
Logistic regression						
−1	0.877	0.821	0.810	0.013	0.791	0.034
−2	0.843	0.718	0.863	0.020	0.757	0.096
−3	0.833	0.779	0.784	0.017	0.821	0.075
−4	0.822	0.792	0.757	0.016	0.835	0.073
−5	0.815	0.792	0.761	0.016	0.833	0.070
−6	0.812	0.649	0.853	0.024	0.789	0.134
−7	0.812	0.645	0.859	0.024	0.785	0.131
−8	0.794	0.649	0.822	0.024	0.825	0.114
−9	0.789	0.757	0.695	0.020	0.873	0.051
−10	0.774	0.611	0.868	0.025	0.791	0.118
−11	0.777	0.696	0.813	0.020	0.831	0.078
−12	0.788	0.735	0.750	0.019	0.863	0.043
−13	0.796	0.731	0.800	0.018	0.837	0.102
−14	0.788	0.698	0.794	0.019	0.855	0.067
−15	0.797	0.629	0.866	0.021	0.812	0.124
−16	0.811	0.783	0.762	0.013	0.865	0.061
−17	0.815	0.679	0.811	0.017	0.862	0.078
−18	0.806	0.545	0.934	0.021	0.735	0.193
−19	0.804	0.704	0.840	0.015	0.841	0.092
−20	0.543	0.132	0.955	0.037	0.891	0.000
−21	0.513	0.038	0.987	0.039	0.889	0.000
−22	0.807	0.760	0.781	0.012	0.878	0.029
−23	0.841	0.898	0.710	0.006	0.891	0.024
−24	0.805	0.778	0.742	0.011	0.901	0.048
Mean (SD)						
Testing data with missing values						
BARTm						
−1	0.882	0.832	0.786	0.018	0.752	0.105
−2	0.859	0.772	0.805	0.026	0.731	0.112
−3	0.818	0.868	0.607	0.020	0.829	0.051
−4	0.836	0.784	0.774	0.025	0.757	0.104
−5	0.857	0.811	0.772	0.022	0.753	0.096
−6	0.859	0.903	0.667	0.013	0.803	0.066
−7	0.859	0.876	0.685	0.016	0.799	0.071
−8	0.840	0.733	0.815	0.029	0.735	0.119
−9	0.823	0.828	0.668	0.021	0.828	0.056
−10	0.827	0.759	0.745	0.027	0.798	0.096
−11	0.816	0.737	0.772	0.028	0.784	0.103
−12	0.820	0.828	0.640	0.022	0.839	0.049
−13	0.833	0.754	0.747	0.027	0.801	0.087
−14	0.832	0.890	0.616	0.014	0.846	0.052
−15	0.802	0.764	0.688	0.026	0.838	0.068
−16	0.820	0.750	0.749	0.023	0.824	0.079
−17	0.850	0.797	0.722	0.021	0.821	0.071
−18	0.845	0.883	0.650	0.013	0.842	0.048
−19	0.835	0.802	0.731	0.020	0.817	0.070
−20	0.825	0.783	0.737	0.021	0.825	0.077
−21	0.826	0.849	0.693	0.016	0.830	0.052
−22	0.844	0.839	0.681	0.016	0.846	0.057
−23	0.844	0.811	0.731	0.017	0.828	0.069
−24	0.829	0.809	0.713	0.018	0.839	0.056
Mean (SD)						
Validation data with missing values						
BARTm						
−1	0.892	0.836	0.790	0.016	0.764	0.105
−2	0.872	0.819	0.770	0.018	0.784	0.097
−3	0.836	0.868	0.772	0.020	0.757	0.051
−4	0.855	0.839	0.713	0.017	0.818	0.072
−5	0.856	0.810	0.751	0.019	0.801	0.086
−6	0.864	0.814	0.779	0.180	0.781	0.112
−7	0.858	0.782	0.784	0.020	0.787	0.112
−8	0.844	0.810	0.739	0.019	0.813	0.102
−9	0.827	0.775	0.757	0.021	0.812	0.107
−10	0.820	0.714	0.797	0.024	0.803	0.116
−11	0.822	0.748	0.766	0.021	0.824	0.092
−12	0.836	0.699	0.821	0.023	0.798	0.109
−13	0.831	0.754	0.766	0.020	0.832	0.108
−14	0.832	0.746	0.773	0.019	0.837	0.100
−15	0.821	0.791	0.745	0.016	0.846	0.082
−16	0.819	0.817	0.703	0.015	0.864	0.078
−17	0.828	0.742	0.788	0.018	0.840	0.095
−18	0.831	0.720	0.827	0.018	0.818	0.120
−19	0.831	0.737	0.799	0.017	0.841	0.110
−20	0.823	0.796	0.729	0.014	0.869	0.076
−21	0.816	0.667	0.830	0.020	0.835	0.118
−22	0.831	0.794	0.737	0.013	0.872	0.075
−23	0.845	0.714	0.837	0.016	0.827	0.113
−24	0.815	0.722	0.793	0.015	0.867	0.080
Mean (SD)						

*AUC*, Area under the receiver operating characteristic curve; *PPV*, positive predictive value; *NPV*, negative predictive value; *BARTm*, bootstrap aggregated regression trees machine; *SD*, standard deviation; *AKI*, acute kidney injury; *ICU*, intensive care unit.

**Table E7:** Mean predicted probabilities for both models for each lead time and actual proportion of patients with AKI, based on each experiment

Dataset	Lead time	Mean predicted probability (SD)		AKI (%)
		Logistic Regression	BARTm	
Train (complete)	−1	9.26 (19.48)	9.11 (14.52)	9.26
	−2	8.78 (16.99)	8.70 (12.72)	8.78
	−3	8.79 (16.29)	8.70 (12.54)	8.79
	−4	8.79 (16.07)	8.71 (12.01)	8.79
	−5	8.79 (16.38)	8.75 (12.67)	8.79
	−6	8.87 (15.64)	8.83 (12.12)	8.87
	−7	8.87 (15.78)	8.76 (12.16)	8.87
	−8	8.68 (16.02)	8.71 (12.23)	8.68
	−9	9.01 (16.31)	8.92 (13.12)	9.01
	−10	8.78 (16.33)	8.75 (12.49)	8.78
	−11	8.36 (15.73)	8.31 (12.57)	8.36
	−12	8.14 (16.02)	8.11 (11.88)	8.14
	−13	7.88 (14.73)	7.81 (10.68)	7.88
	−14	7.90 (14.16)	7.91 (10.56)	7.90
	−15	7.55 (13.65)	7.58 (10.48)	7.55
	−16	7.69 (13.99)	7.73 (10.30)	7.69
	−17	7.29 (13.40)	7.31 (9.65)	7.29
	−18	7.18 (12.87)	7.22 (9.69)	7.18
	−19	6.97 (13.30)	6.96 (9.75)	6.97
	−20	5.13 (22.06)	7.17 (10.09)	7.18
	−21	0.78 (8.80)	6.81 (10.27)	6.86
	−22	7.03 (13.12)	7.02 (10.00)	7.03
	−23	6.88 (13.62)	6.84 (10.17)	6.88
	−24	6.33 (12.13)	6.39 (9.22)	6.33
Test (complete)	−1	8.75 (19.32)	8.71 (13.72)	8.39
	−2	8.69 (17.47)	8.28 (12.12)	9.37
	−3	9.15 (17.29)	8.70 (12.54)	9.29
	−4	9.19 (17.11)	8.61 (11.45)	9.29
	−5	8.97 (16.90)	8.69 (11.67)	9.29
	−6	9.40 (16.44)	8.60 (11.97)	9.05
	−7	9.42 (16.53)	8.50 (11.43)	9.05
	−8	8.64 (15.89)	8.52 (12.03)	9.15
	−9	8.77 (16.17)	8.46 (11.81)	8.18
	−10	8.96 (16.81)	8.53 (11.48)	8.44
	−11	8.58 (16.41)	7.87 (9.98)	8.54
	−12	7.19 (15.13)	7.19 (11.04)	8.31
	−13	7.57 (13.62)	7.89 (10.54)	8.33
	−14	7.57 (13.82)	7.63 (9.86)	7.78
	−15	6.89 (12.11)	7.11 (9.49)	6.88
	−16	6.86 (12.69)	7.21 (9.54)	7.48
	−17	7.20 (13.70)	7.19 (9.30)	7.32
	−18	6.96 (13.04)	6.92 (9.06)	7.58
	−19	6.88 (13.14)	6.68 (9.63)	7.00
	−20	4.17 (20.00)	7.16 (9.53)	7.01
	−21	1.09 (10.37)	6.48 (9.46)	7.27
	−22	6.84 (13.34)	6.77 (9.54)	6.77
	−23	7.30 (15.21)	6.68 (8.73)	6.61
	−24	6.62 (12.87)	6.18 (8.58)	6.81
Test (NA)	−1	–	9.24 (13.76)	7.83
	−2	–	8.48 (11.80)	8.51
	−3	–	8.70 (12.54)	8.45
	−4	–	9.20 (12.73)	8.45
	−5	–	8.74 (12.07)	8.45
	−6	–	8.84 (11.59)	8.29
	−7	–	8.75 (11.50)	8.29
	−8	–	8.50 (11.42)	8.34
	−9	–	7.73 (11.19)	7.66
	−10	–	9.00 (12.09)	7.83
	−11	–	8.68 (11.84)	7.85
	−12	–	7.41 (10.70)	7.69
	−13	–	7.95 (10.25)	7.69
	−14	–	7.55 (9.59)	7.29
	−15	–	7.12 (8.40)	7.31
	−16	–	7.03 (9.32)	6.69
	−17	–	7.04 (9.03)	7.09
	−18	–	6.73 (8.92)	6.93
	−19	–	6.98 (9.37)	7.00
	−20	–	7.42 (9.74)	6.65
	−21	–	6.59 (9.48)	6.88
	−22	–	7.03 (9.75)	6.48
	−23	–	6.76 (9.59)	6.43
	−24	–	5.99 (8.39)	6.39
Validation (complete)	−1	5.68 (15.11)	7.32 (12.09)	5.77
	−2	6.83 (15.30)	7.36 (10.72)	5.77
	−3	7.98 (15.63)	8.03 (10.97)	5.70
	−4	8.34 (15.23)	8.03 (10.63)	5.70
	−5	8.32 (15.56)	7.81 (11.87)	5.70
	−6	8.67 (15.71)	8.48 (11.07)	5.70
	−7	8.30 (15.09)	8.04 (10.26)	5.63
	−8	8.41 (15.50)	8.18 (11.09)	5.51
	−9	7.84 (14.34)	8.30 (10.35)	5.52
	−10	7.10 (14.45)	7.30 (10.31)	5.38
	−11	7.13 (14.53)	7.15 (9.56)	5.18
	−12	5.61 (12.41)	6.96 (10.39)	5.11
	−13	8.62 (15.31)	7.71 (9.59)	5.04
	−14	6.07 (12.11)	7.65 (9.23)	4.76
	−15	7.12 (13.04)	7.15 (9.17)	4.69
	−16	6.60 (12.83)	7.37 (9.60)	4.54
	−17	6.33 (12.05)	5.97 (7.59)	4.25
	−18	6.35 (12.22)	6.56 (8.02)	4.18
	−19	6.63 (13.23)	6.06 (8.35)	4.11
	−20	4.89 (21.57)	6.70 (8.50)	4.05
	−21	1.38 (11.66)	6.12 (7.96)	3.98
	−22	3.75 (10.24)	5.90 (8.02)	3.85
	−23	5.14 (12.63)	6.05 (8.55)	3.81
	−24	5.74 (11.34)	5.53 (7.53)	3.53
Validation (NA)	−1	–	9.20 (14.11)	7.21
	−2	–	8.59 (11.70)	7.21
	−3	–	8.70 (12.74)	7.13
	−4	–	8.40 (11.92)	7.09
	−5	–	8.94 (12.62)	7.09
	−6	–	9.21 (11.61)	7.05
	−7	–	8.96 (11.14)	6.97
	−8	–	9.68 (12.04)	6.89
	−9	–	9.12 (11.78)	6.77
	−10	–	8.05 (10.68)	6.54
	−11	–	7.81 (10.43)	6.26
	−12	–	7.25 (9.67)	6.10
	−13	–	8.57 (10.32)	5.90
	−14	–	8.02 (9.20)	5.57
	−15	–	6.93 (7.85)	5.53
	−16	–	7.85 (9.78)	5.41
	−17	–	6.94 (8.53)	5.17
	−18	–	7.20 (8.67)	5.09
	−19	–	7.46 (9.41)	4.92
	−20	–	7.30 (9.30)	4.88
	−21	–	6.35 (8.76)	4.80
	−22	–	6.69 (8.77)	4.63
	−23	–	6.55 (8.96)	4.55
	−24	–	5.65 (7.83)	4.22

*SD*, Standard deviation; *AKI*, acute kidney injury; *BARTm*, bootstrap aggregated regression trees machine.

## Appendix E1 Model Development Methods

This study gained ethical approval from the responsible UK Health Research Authority (REC18/YH/0366, September 21, 2018). Since this was a retrospective analysis of routinely collected clinical data, the requirement for written informed consent was waived by the institutional review board. The article adheres to the Transparent Reporting of a multivariable prediction model for Individual Prognosis or Diagnosis guidelines.^E1^

In this paper, 2 models were developed to predict the onset of acute kidney injury (AKI) within 25 hours since admission to the intensive care unit (ICU) on an hourly basis, up to 24 hours in advance, will be presented. These models are logistic regression (LR) and bootstrap aggregated regression trees machine (BARTm). (As part of this study, other methods were also experimented with, the details and results of which can be found from https://stax.strath.ac.uk/concern/theses/6969z130f.)

### Predicted Outcome

The Kidney Disease” Improving Global Outcomes clinical practice guideline^E2^ was used to define AKI. Retrospective diagnosis was given, by dividing each serum creatinine level, measured in the ICU, by the preoperatively measured serum creatinine level (baseline), as done by Birnie and colleagues.^E3^ Ff the difference was greater than or equal to 1.5 times the baseline, the patient was diagnosed to have AKI. In addition, the timestamp when the creatinine difference occurred was recorded as a timestamp to indicate the occurrence of AKI. The urine output was not used to diagnose AKI, as per the Kidney Disease: Improving Global Outcomes definition, because the urine output has been shown to be overly sensitive and nonspecific for the cardiac surgery population.^E4^

### Setting and Datasets

This study was conducted at the Golden Jubilee National Hospital, a large cardiac center in the United Kingdom that performs more than 50% of all elective cardiothoracic surgeries for the National Health Service in Scotland.^E5^ Data from 2 local electronic health record databases were used: the Cardiac, Cardiology and Thoracic Health Information (CaTHI) database, which includes static information recorded preoperatively, and the Centricity CIS Critical Care database, which includes dynamic laboratory data from the ICU. Data for patients undergoing coronary artery bypass graft, aortic valve, and combined coronary artery bypass graft and valve surgeries between April 1, 2012, and December 31, 2018, were included for the development phase (training and testing) of the models. The patient data between January 1, 2019, and June 30, 2022, were used to internally validate the models. Only the records that occurred in the dataset for the patient for the first time (unique entries) were included in the analysis. Furthermore, patients who experienced AKI within the first hour since ICU admission were excluded due to no laboratory data available for these patients to allow for prediction of AKI. Finally, of patients with AKI, only those who had AKI within the first 25 hours were observed, as the majority of patients were diagnosed with AKI within that time frame (as shown in the Results). Thus, the final number of patients included in this study was 6056 patients for development and 3572 patients for validation. The derivation of training and testing datasets and how the final number of patients was arrived at is further described in “Missing Data” section.

### Predictors

In total, 82 variables were used in the models, including 25 preoperatively recorded variables, including demographic variables (eg, sex and age), information about the surgery (eg, type and urgency of the surgery), and comorbidities, relevant to cardiac surgery (eg, cardiac, neurologic, renal, and respiratory function). From the ICU database, 13 laboratory variables and 4 medicine-related variables were included. The full list of variables included in the models, together with descriptive statistics can be found from Table E1.

The preoperative variables were measured only once at the preoperative assessment clinic, and were therefore treated as static variables in the models. The laboratory variables were measured repeatedly, allowing for the development of an hourly prediction model.

As shown in Table 1, each laboratory variable was measured at different times, depending on patient's needs. Furthermore, there is a significant difference between the frequencies of how often the laboratory variables are measured, when comparing the development data and validation data. It is especially noticeable that in the (more recent) validation dataset, measures of creatinine, urea and C-reactive protein are made about every 6 hours, whereas in the development dataset, these variables are measured every 10 hours.

### Missing Data

For preoperative data from the CaTHI database, patients who had not been discharged from the hospital by the time of data extraction were excluded from the analysis due to not having their final outcome recorded (ie, deceased or discharged). Patients with “salvage” priority, and “unknown” New York Heart Association grade, previous myocardial infarction, and hypertension history were excluded due to very small group of patients not having these variables recorded for them. The cases with many “unknown” entries for categorical variables were included in the analysis and left coded as “unknown.”

Also, because some of the validation data was recorded during the start of the coronavirus disease 2019 (COVID-19) pandemic, due to the lack of understanding regarding the specific effects of past COVID-19 infection on surgical outcomes, patients who were indicated to have past COVID-19 or COVID-19 infection at the time of ICU in the clinicians’ notes were excluded from the analysis (1122 patients). For numerical variables, patients with clinically infeasible values were excluded. If a numerical variable was recorded for less than 80%^E6^ of the patients, the variable was excluded from analysis. The only variable excluded for that reason was preoperative hemoglobin level.

Although the CaTHI database is an audit database, and therefore should include fewer “unknowns,” the only variables that are mandatory in the CaTHI database are those needed to calculate the logistic EuroSCORE.^E7^ Therefore, until a study was conducted in 2016, using this database, the information regarding New York Heart Association grade, previous myocardial infarction, and hypertension history and hemoglobin was not always recorded. Furthermore, as this information is usually recorded at the preoperative assessment clinic, medical history for patients with emergency surgery might not always be readily available.

The ICU data from Centricity CIS database was checked for obvious incorrect values, in case of which the values were marked as NA. If a patient had a timestamp recorded for a missing value of a variable, the previously recorded value was carried forward to the next timestamp. The only variable recorded for all ICU patients was hemoglobin, with 100% completeness. Creatinine was recorded for almost all patients, with 98.32% completeness. Instead of using medicine doses, since these were recorded for less than 41.00% of the patients, medicine variables were recorded as binary categorical variables based on whether the patient was given medication (yes vs no).

### Classification Methods and Experiments

In the development phase of the study, other classification methods were also experimented with (see Lapp^E8^); however, LR and BARTm were chosen for the validation of the study due to logistic regression's high interpretability and competitiveness with other classification methods^E9^ and BARTm's ability to incorporate missing values into its prediction.^E10^

Although missing data are a common problem in electronic health records,^E11^ incorporating classification methods that can handle missing data without imputation methods which could lead to bias^E12^ is rare.^E13^ BARTm is a method that can handle missing values by incorporating built-in estimates of uncertainty in the form of credible intervals as well as previous information on covariates. This is done by sending missing data to whichever of the 2 daughter nodes increases the overall model likelihood.^E14^ This means, if we have 2 options (eg, left and right), then BARTm offers options for both of these paths if a record has a missing value. Hence, there is a consideration that the direction of missingness is equally likely to be left or right, conditional on the splitting attribute and value.^E10^ It has been previously shown that BARTm is comparable with the performance of random forest with missForest imputation.^E15^

The models were developed on a complete set of training data (ie, all records including missing values were removed). To take advantage of being able to incorporate missing values into the prediction model, 2 experiments were undertaken in terms of incorporating missing values to testing and validation sets.

Experiment 1: Testing and validating the models using complete data (ie, removing all records that included missing values). The results of LR and BARTm are presented.

Experiment 2: Testing and validating the models on datasets that included some missing values. Records with >40% of missing values were excluded from analysis, as done elsewhere.^E16^ The rest of the missing values were left as is. Here, the results of BARTm are presented since this method is robust to handle missing data.^E10^

For logistic regression, the *“caret”* R package version 6.0.93 with method “glm” was used.^E17^ For BARTm, the *“bartMachine”* package,^E18^ version 1.3.3.1, in R was used, together with the default of including missing values as the model can accommodate these. The models were developed on the training data, using 10-fold cross validation (The general code for developing the classification models can be found from https://doi.org/10.15129/1ab360f7–0779–4cf3–8a9a–dae621892a51), which is the recommended approach to developing a prediction model.^E1^ All analysis was conducted, using R, version 4.2.2.

### Data Preparation and Time Windows

The models were developed to predict AKI on an hourly basis. To facilitate this, rolling time windows were created to first indicate the onset of the predicted outcome. Second, the time windows were used to develop prediction models for each time window before the event. In this study, the hourly prediction was undertaken for AKI within 25 hours of ICU stay. This timeframe was chosen because the majority of patients experienced the onset of AKI between 20 and 30 hours since ICU admission, as shown in the section “Patient Population and Acute Kidney Injury.”

The models predicting AKI were built for hourly lead times, based on the time windows. The lead times were chosen to be every hour from 1 to 24 hours ahead of the onset of AKI occurring within 25 hours since ICU admission. For example, if predicting AKI 1 hour in advance, the data were collected from the admission to ICU until 1 hour before the onset of AKI. In general terms, the model predicting AKI at lead time n used all data that were collected until n hours before the outcome, which is also illustrated in Figure 5 in the main article.

To simplify the laboratory data used in models, for each laboratory variable for the minimum, maximum, and first and last measurement for each lead time were used, helping to create a more consistent set of input data for the models, which might otherwise have had to deal with variations in the number of independent variables at each stage (shown in Figure E1).^E19^^,^^E20^ This means that if the predicted outcome happened in time window = 6, for each variable first, last, min, and max measurements that occurred in time windows 0 to 5 were calculated. Regardless of the number of hours after admission that AKI occurred, there would always be 4 values (first, last, min and max) for each dynamic predictor variable.

The prediction models had a binary outcome (AKI = yes/no), but only patients with AKI = yes had a timestamp associated with the outcome recorded. Hence, an arbitrary time as the end point was chosen for patients with AKI = no. Most patients had AKI between 20 and 25 hours since ICU admission and so an arbitrary end point of 25 hours for AKI prediction was chosen.

Finally, it is important to note that patients who had the predicted outcome recorded within the first hour since ICU admission were removed from analysis, as done in other predictive modeling studies.^E20^ This is because the hourly prediction model is intended to be used in the ICU, and hence it is impossible to predict an outcome that has already happened. Hence, 545 patients who had AKI on admission to the ICU were excluded from the analysis in the development dataset and 309 patients from validation dataset.

For both logistic regression and BARTm models, all available variables were included, and the time points were treated as independent, ie, the predictions carried out by the models were not dependent on the previous time points.

### Training, Testing, and Validation Data

To develop the models, the datasets for each lead time were divided into a training set (2/3 of data) and testing set (1/3 of data). For every experiment, the models developed using the training data that did not contain any missing values. As explained previously, the models were tested and validated on both complete data and data with missing values.

Due to AKI occurring at different times for patients in the ICU, the number of patients in each dataset—and consequently the number of data points used in the creation of the model—in each lead time is different (shown in Table E1). Since the same training data was used for both experiments, the mean number of patients in training data was 2464 (standard deviation [SD] = 25.75). The mean number of patients in testing data was 1212 (SD = 12.96) and 1740 (SD = 9.75) for Experiments 1 and 2, respectively. For validation data, the mean number of patients was 1327 (SD = 21.78) for Experiment 1 and 2341 (25.18) for Experiment 2. As explained earlier, the training data was complete for both experiments, and the testing and validation datasets were complete for Experiment 1. For Experiment 2 (with the missing data) the mean completeness across all lead times was 96.96% (SD = 0.04) for testing data and 96.61% (SD = 0.06) for validation data.

### Performance Measures

The models' performance measures were calculated for training, testing and validation sets for each experiment for each lead time. The models were evaluated based on discrimination, ie, the area under the receiver operating characteristic curve, sensitivity, specificity, and positive predictive value and negative predictive value. The performance metrics were calculated, using the optimal cut-off points, where sensitivity and specificity were maximized. In this article, mean and SD for each performance measure across all lead times are presented. The models’ performance measures across all lead times were compared using *t* tests with the significance level set to .05.

To understand the applicability of the models in this specific patient population, the models’ calibration was assessed, using calibration plots and predicted versus observed probabilities for AKI. As the models developed in this paper were only validated internally, in case of poor calibration, the models were not recalibrated as the average of predicted risks would match the event rate.^E21^
